# Titanium dioxide particles frequently present in face masks intended for general use require regulatory control

**DOI:** 10.1038/s41598-022-06605-w

**Published:** 2022-02-15

**Authors:** Eveline Verleysen, Marina Ledecq, Lisa Siciliani, Karlien Cheyns, Christiane Vleminckx, Marie-Noelle Blaude, Sandra De Vos, Frédéric Brassinne, Frederic Van Steen, Régis Nkenda, Ronny Machiels, Nadia Waegeneers, Joris Van Loco, Jan Mast

**Affiliations:** 1 Trace Elements and Nanomaterials, Sciensano, Groeselenbergstraat 99, 1180 Uccle, Belgium; 2 Trace Elements and Nanomaterials, Sciensano, Leuvensesteenweg 17, 3080 Tervuren, Belgium; 3 Service Risk and Health Impact Assessment, Sciensano, Juliette Wytsmanstraat 14, 1050 Brussels, Belgium

**Keywords:** Environmental, health and safety issues, Nanometrology, Nanoparticles

## Abstract

Although titanium dioxide (TiO_2_) is a suspected human carcinogen when inhaled, fiber-grade TiO_2_ (nano)particles were demonstrated in synthetic textile fibers of face masks intended for the general public. STEM-EDX analysis on sections of a variety of single use and reusable face masks visualized agglomerated near-spherical TiO_2_ particles in non-woven fabrics, polyester, polyamide and bi-component fibers. Median sizes of constituent particles ranged from 89 to 184 nm, implying an important fraction of nano-sized particles (< 100 nm). The total TiO_2_ mass determined by ICP-OES ranged from 791 to 152,345 µg per mask. The estimated TiO_2_ mass at the fiber surface ranged from 17 to 4394 µg, and systematically exceeded the acceptable exposure level to TiO_2_ by inhalation (3.6 µg), determined based on a scenario where face masks are worn intensively. No assumptions were made about the likelihood of the release of TiO_2_ particles itself, since direct measurement of release and inhalation uptake when face masks are worn could not be assessed. The importance of wearing face masks against COVID-19 is unquestionable. Even so, these results urge for in depth research of (nano)technology applications in textiles to avoid possible future consequences caused by a poorly regulated use and to implement regulatory standards phasing out or limiting the amount of TiO_2_ particles, following the safe-by-design principle.

## Introduction

Wearing face masks is an important and widely applied public health measure to control the COVID-19 pandemic^1^. A recent study, testing several batches of face masks intended to be put on sale as personal protective equipment, showed that 70% of the examined face masks contained TiO_2_ in quantities ranging from 100 to 2000 mg kg^−1^^2^. This suggests that TiO_2_ is commonly applied in textiles of face masks, as in a wide variety of other textiles, e.g. to improve stability to ultraviolet light, as white colorant or as a matting agent^3,4^. In addition, to introduce new solutions to the challenges associated with the COVID-19 pandemic, textile companies are incorporating specific nanofiber, nanocomposite and nanoparticle technology into face masks^5,6^. Nanofibers containing TiO_2_ nanoparticles have been produced to create antimicrobial filters^7^, also in combination with silver^8^ and graphene^9^. Coatings of TiO_2_ nanoparticles on cotton fabric were applied for enhanced self-cleaning and antibacterial properties^10^.

In their recent opinion paper, Palmeiri et al.^5^ warn for the possible future consequences caused by a poorly regulated use of nanotechnology in textiles applied to improve the performance of face masks. In animal experiments, toxic effects were reported when TiO_2_ particles were inhaled^11,12^, as well as when they were ingested orally^13,14^. In 2017, the Risk Assessment Committee (RAC) of the European Chemical Agency (ECHA) reviewed the carcinogenic potential of TiO_2_ and proposed to classify Titanium dioxide as Carc. 2, H351 (suspected human carcinogen)^15^ by inhalation. This CLP classification^16^ was adopted for titanium dioxide.

To evaluate whether the TiO_2_ particles in face masks possibly present a health risk, their amounts, their physicochemical properties and their localization were analyzed in a selection of face masks. Supporting on these measurements, the amount of TiO_2_ at the surface of the textile fibers was estimated and compared with the acceptable exposure level to TiO_2_ by inhalation, expressed per mask (AEL_mask_).

## Results and discussion

Twelve face masks meant to be worn by the general population and including both single-use (disposable) and re-usable masks were obtained from various suppliers in Belgium and the EU. The origin of the masks is worldwide. The selected masks consist of a variety of fibers, including synthetic fibers, such as polyester, polyamide and meltblown and thermobonded non-woven fabrics; and natural fibers, such as cotton (Table 1). All masks are NIOSH uncertified, Mask04 and Mask07 have a CE logo; Mask03 and Mask07 are OEKO-TEX certified. Mask01, 04 and 05 are three ply type masks^17,18^. Images of the examined masks are given as Supplementary Information 1.

**Table 1:** Properties of the examined face masks.

Ref	Type^a^	Layers	Composition^a^	Fiber diameter^b^ (µm)	AA size^c^ (nm)	CP size^d^ (nm)	Modelled fraction of TiO_2_ on fiber surface (%)	Total TiO_2_ per mask^f^ (µg)	Estimated amount of TiO_2_ at fiber surface per mask (µg)	Times the amount of TiO_2_ at fiber surface exceeds AEL_mask_
Mask01	Single-use	External	Thermobonded non-woven^e^	24 ± 7.4	197 ± 59	131 ± 61	2	2386 ± 286	39	11
		Central	Meltblown non-woven	–	–	–	–			
		Internal	Thermobonded non-woven	–	–	–	–			
Mask02	Reusable	External	Polyester^e^	9 ± 0.7	123 ± 76	125 ± 72	3	17,332 ± 2080	462	128
		Central	Meltblown non-woven	–	–	–	–			
		Internal	100% cotton	–	–	–	–			
Mask03	Reusable	External	100% polyester^e^	9 ± 0.9	188 ± 121	124 ± 48	4	30,757 ± 3691	1056	293
		Central	65% polyester, 35% cotton^e^	11 ± 0.7	156 ± 104	107 ± 59	3			
		Internal	65% polyester, 35% cotton^e^	11 ± 2.0	173 ± 101	133 ± 45	3			
Mask04	Single-use	External	Thermobonded non-woven^e^	21 ± 1.8	220 ± 66	182 ± 80	2	1370 ± 164	30	8
		Central	Meltblown non-woven	–	–	–	–			
		Internal	Thermobonded non-woven^e^	23 ± 7.2	274 ± 76	143 ± 97	2			
Mask05	Single-use	External	Thermobonded non-woven^e^	19 ± 2.5	233 ± 107	184 ± 74	2	791 ± 95	17	5
		Central	Meltblown non-woven	–	–	–	–			
		Internal	Thermobonded non-woven^e^	18 ± 2.8	171 ± 82	168 ± 58	2			
Mask06	Reusable	External	Polyester^e^	7 ± 0.4	147 ± 57	101 ± 46	4	12,195 ± 1463	448	87
		Central	Unknown^e^	35 ± 8.4	409 ± 182	117 ± 32	2			
		Internal	Cotton	–	–	–	–			
Mask07	Reusable	External	Polyamide^e^	9 ± 1.2	130 ± 98	96 ± 38	3	152,345 ± 18,281	4394	1220
		Internal	Polyamide^e^	9 ± 0.7	135 ± 118	103 ± 44	3			
Mask08	Reusable		Polyester, polyamide, elastane^e^	11 ± 1.0	184 ± 87	99 ± 50	3	9573 ± 1149	327	136
Mask09	Reusable	External	Thermobonded non-woven^e^	24 ± 6.5	450 ± 130	147 ± 73	4	2298 ± 276	84	16
		Central	Meltblown non-woven	–	–	–	–			
		Internal	Thermobonded non-woven^e^	23 ± 6.4	372 ± 177	157 ± 56	3			
Mask10	Reusable	External	Polyester^e^	11 ± 0.8	110 ± 84	90 ± 40	2	17,427 ± 2091	358	99
		Internal	Cotton	–	–	–	–			
Mask11	Reusable	External	Polyester^e^	10 ± 0.6	135 ± 69	89 ± 32	3	12,713 ± 1526	352	98
		Central	Thermobonded non-woven	–	–	–	–			
		Internal	Cotton	–	–	–	–			
Mask12	Reusable	External	Bi-component microfiber^e^	13 ± 0.8	173 ± 95	105 ± 45	8	12,929 ± 1552	1054	293
		Internal	Bi-component microfiber^e^	12 ± 1.7	191 ± 104	96 ± 43	9			

^a^As indicated on the packaging or based on expert advice.

^b^Median ± interquartile range (IQR) of the fiber diameter.

^c^Minimum external dimension of the TiO_2_ agglomerates (AA) estimated as the median ± interquartile range (IQR) of the minimum Feret diameter distribution.

^d^Minimum external dimension of the TiO_2_ constituent particles (CP) estimated as the median ± IQR of the minimum Feret diameter distribution.

^e^TiO_2_ particles were observed in this layer by STEM-EDX.

^f^The combined measurement uncertainty (k = 1) on the total mass of TiO_2_ per mask was calculated based on an in-house validation study (see Supplementary Information 8). The uncertainty related to the weight of the mask is not included in this uncertainty.

Measurement of the total amount of titanium (Ti) in each face mask, as a proxy for the amount of TiO_2_ particles, by inductively coupled plasma-optical emission spectroscopy (ICP-OES) showed that the amount of TiO_2_ varied strongly, from 0.8 to 152 mg per mask (Table 1).

**Figure 1:**
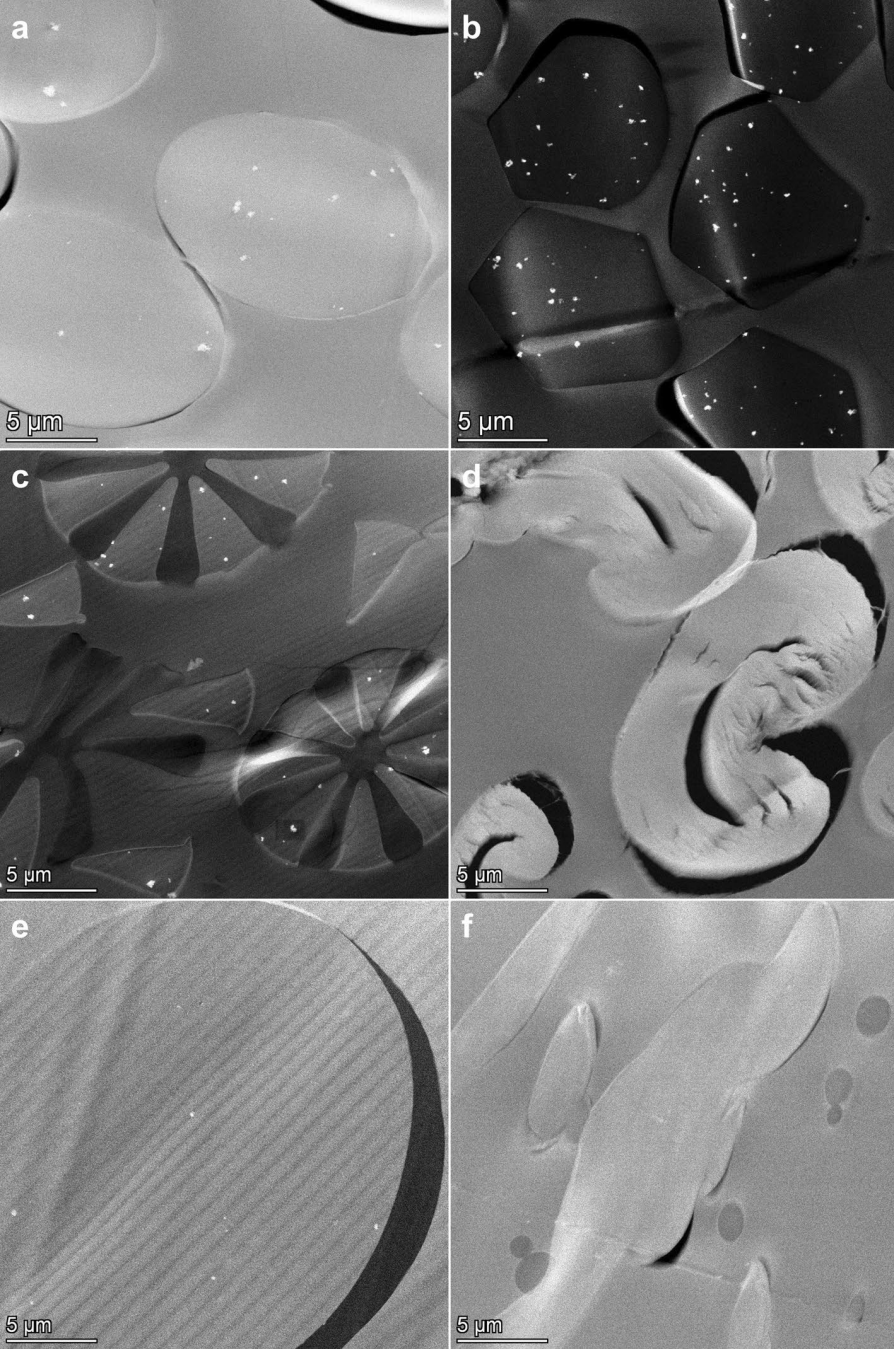
HAADF- STEM images of sections of different types of fibers observed in the face masks, with (**a**) polyester, (**b**) polyamide, (**c**) bi-component microfiber, (**d**) cotton, (**e**) thermobonded non-woven fiber, (**f**) meltblown non-woven fabric. TiO_2_ particles are visible as bright white dots.

**Figure 2:**
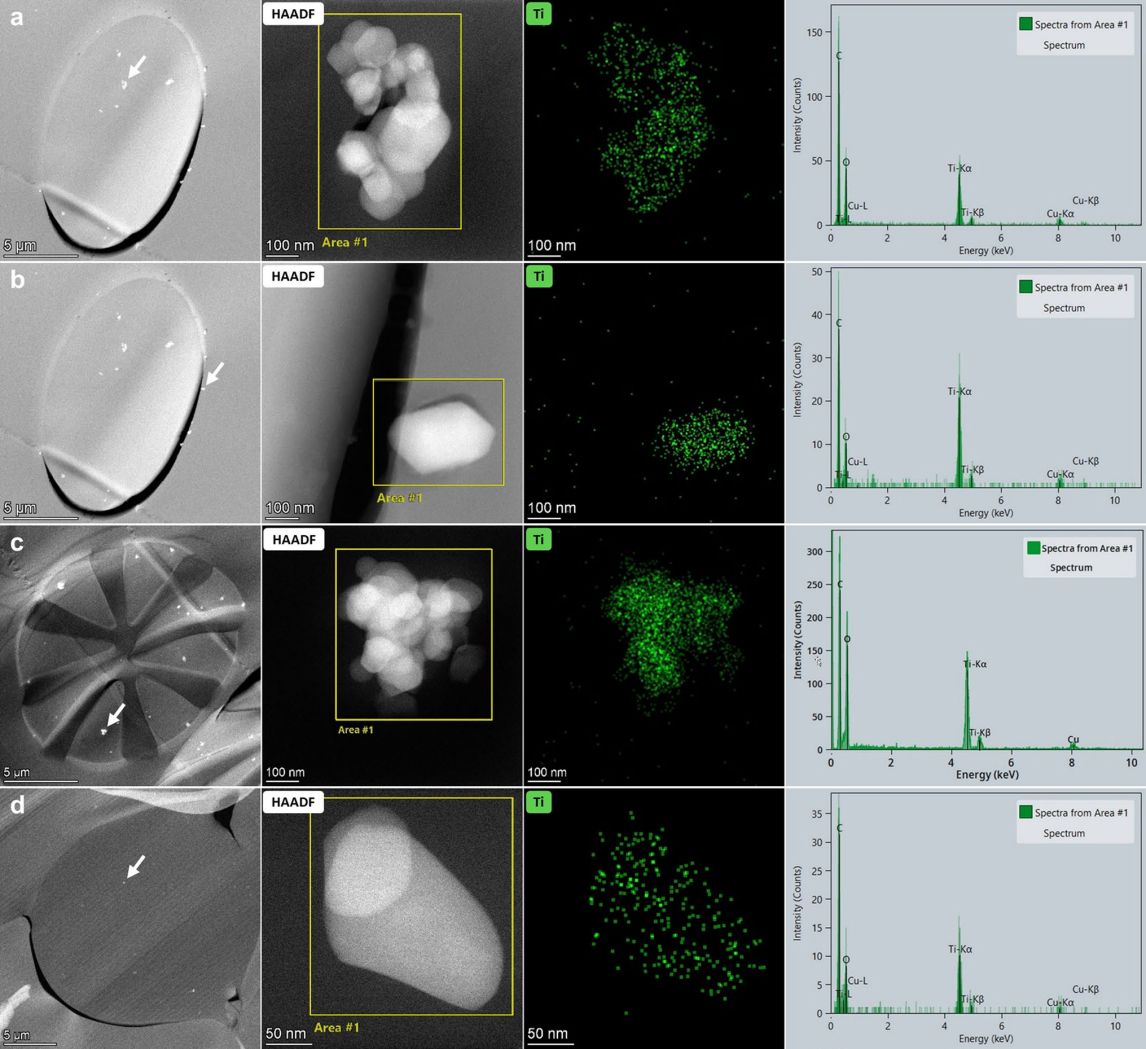
STEM-EDX analysis of particles (**a**) in a polyester fiber, (**b**) at the edge of a polyester fiber, (**c**) in a bi-component microfiber and (**d**) in a non-woven fabric. (first column) The low magnification HAADF-STEM images show the cross sections of the fibers containing the analyzed particles (white arrows) shown in the (second column) higher magnification HAADF-STEM images. (third column) The spectral images of Ti (green) obtained by EDX show that the measured Ti signal coincides with the position of the particles shown in the STEM image, and (fourth column) the EDX spectra of the area’s indicated on the STEM image show the Ti signal.

High angle annular dark field (HAADF)-scanning transmission electron microscopic (STEM) analysis of sections of the resin embedded face masks showed single and agglomerated constituent particles in synthetic fibers (Fig. 1a–c,e). The particles were observed in at least one layer of each examined face mask (Table 1 and Supplementary Information 2). Energy dispersive X-ray spectroscopy (EDX) analysis confirmed that these particles consist of TiO_2_ (Fig. 2 and Supplementary Information 3). A fraction of the TiO_2_ particles was located at the surface of the fibers (Fig. 2b). TiO_2_ particles were not observed in cotton fibers (Fig. 1d), in meltblown non-woven fabrics (Fig. 1f), and in some of the thermobonded non-woven fabrics (Table 1).

In general, the electron microscopy results confirm the ICP-OES measurements showing that the amount of TiO_2_ particles was approximately a factor 10 lower in non-woven fabrics than in polyester and polyamide fibers.

Measurements of the size and shape (near-spherical morphology) of the constituent TiO_2_ particles and agglomerates in the examined face masks (Table 1, Supplementary Information 4–6) show that, overall, the physicochemical properties of the TiO_2_ particles in face masks are in agreement with the specifications of so-called fiber-grade TiO_2_ applied in other textiles^19,20^ and are similar to those of the E 171 food additive^14^. Although the measured TiO_2_ size distributions in the face masks do not all qualify the applied TiO_2_ as nanomaterials according to the EC-definition^21^, each examined mask, besides Mask04, contained a notable fraction of nanoparticles (6% to 65%), requesting an appropriate risk analysis.

Because the hazard of inhaled TiO_2_ particles is well documented^11,22,23^, particularly exposure analysis is important for risk analysis. Exposure to TiO_2_ (nano)particles in face masks, depends on their level of release. Migration of agglomerated TiO_2_ particles completely incorporated in the fiber polymers of face masks can be excluded by theoretical considerations: only particles smaller than 5 nm can migrate in the polymers constituting the face masks^24^. Particles at the fiber surface might, however, be released when they are subjected to abrasion or to aerodynamic forces. Direct measurement of released particles is problematic because, to our knowledge, no standardized methods are available to determine whether particles are released from face masks during normal use, and which amount of TiO_2_ is released. It is unknown if particles could be released as single particles, as agglomerates, as pieces of textile fibers containing agglomerates or a combination thereof, altering their fate. Moreover, few literature data are available that provide information on desorption/erosion/abrasion of TiO_2_ particles from TiO_2_-containing fibers^25^. Therefore, an indirect approach was applied comparing the mass of TiO_2_ at the surface of the textile fibers of each mask with the mass of TiO_2_ particles that can be inhaled without adverse effects, expressed per mask (AEL_mask_). This approach does not assume release of all particles at the fiber surface. It merely calculates which fraction of TiO_2_ particles at the fiber surface has to be released to exceed the acceptable exposure level. Because the fate and release mechanisms of particles from face masks are currently unknown, no assumptions were made about the likelihood of the release of particles itself.

AEL_mask_ was estimated to be 3.6 µg using a threshold-based risk characterization for subchronic exposure with an intensive use scenario of face masks by the general adult population as described in Supplementary information 7. Lung inflammation was chosen as critical effect. A no observed adverse effect concentration of 0.5 mg/m^3^ was determined based on the repeated dose inhalation study with rats of Bermudez et al.^12^ The risk was further characterized supporting on the approach to determine the professional acceptable exposure levels to TiO_2_ nanoforms^26^. The intensive use scenario assumed that 2 masks are worn over an 8-h period, with a recommended change of the masks every 4 h^27^.

Furthermore, it was assumed that TiO_2_ particles in the fiber matrix do not migrate and that only particles at the fiber surface can be released. The fraction (%) and the mass (µg) of TiO_2_ particles at the fiber surface, were modelled assuming a homogenous particle distribution in the fibers as described in the methods section and Supplementary Information 9. This assumption is plausible because the TiO_2_ particles are mixed with the fiber matrix during production and was confirmed by HAADF-STEM analysis. For typical (near-)cylindrical synthetic fibers (polyester, polyamide and non-woven), percentages ranged from 2 to 4%. Estimated amounts of TiO_2_ at the fiber surface per mask ranged from 17 to 4394 µg (Table 1). Because the structure of bi-component microfibers (Fig. 1c) results in a larger surface area^28^, a correction factor was introduced resulting in higher percentages of particles at the surface (in the methods section and Supplementary Information 9).

Table 1 shows that for all examined face masks, the amount of TiO_2_ particles at the surface of the textile fibers notably exceeds the AEL_mask_. This systematic exceedance indicates that by applying an approach relying on conservative assumptions while uncertainties regarding hazard and exposure remain (Supplementary Information 7), a health risk cannot be ruled out when face masks containing polyester, polyamide, thermobonded non-woven and bi-component fibers, are used intensively. Exceedance of the AEL_mask_ for reusable face masks is higher (87 to 1220 times) than for single use masks (5 to 11 times), implying that for the reusable masks uptake of only a very small percentage of the particles at the fiber surface may already pose a health risk. Reusable masks typically have higher TiO_2_ amounts in the matrix, have a higher mass (more textile corresponds with more TiO_2_), and have smaller mean fiber diameters than single use masks. For all examined masks, the combined measurement uncertainty (k = 1) on the total mass of TiO_2_ (Table 1, Supplementary Information 8) was larger than AEL_mask_. Consequently, TiO_2_ release in the order of AEL_mask_, measured as the change in TiO_2_ before and after wearing the mask, cannot be demonstrated since it falls within the uncertainty range of the total mass measurement.

Face mask have an important role in the measures against the COVID-19 pandemic^1^. So far, no data are available that indicate that the possible risk associated with the presence of TiO_2_ particles in face masks outweighs the benefits of wearing face masks as protection measure. That is why we do not call for people to stop wearing face masks. However, the warning of Palmeiri et al.^5^ for the possible future consequences caused by a poorly regulated use of nanotechnology in textiles should be extended to face masks where TiO_2_ particles are applied conventionally, as a white colorant or as a matting agent, or to assure durability reducing polymer breakdown by ultraviolet light^3,4^. These properties are not critical for the functioning of face masks, and synthetic fibers suitable for face mask can be produced without TiO_2_^29^ as was observed in the layers of several masks (Table 1). Moreover, uncertainties regarding the genotoxicity of TiO_2_ particles remain^14^. Therefore, these results urge for the implementation of regulatory standards phasing out or limiting the amount of TiO_2_ particles, according to the ‘safe-by-design’ principle.

The applied approach allowed to assess one of the quality parameters of face masks quantitatively: the amount of TiO_2_ at the fiber surface. Such quantitative parameter is important to evaluate the face masks present on the market, to develop product specifications and regulatory standards, and to produce better products.

In the course of this study, we identified several major challenges related to the analysis, characterization and risk assessment of TiO_2_ in face masks, which go beyond the scope of the study: (i) In general, scientific data on the presence of (nano)particles in face masks, their characteristics, the exposure and the risks for the population is limited. (ii) Methodologies for characterizing TiO_2_ particles in face masks are time consuming and expensive. (iii) Even though this study focused on face masks intended for the general public, this does not exclude TiO_2_ from being present in other types of masks containing synthetic fibers, such as medical masks, even when they are certified. The presented study on face masks for the general population should be extended to assess the potential health risks associated with the presence of TiO_2_ particles in medical and personal protection equipment face masks and consequent occupational exposure. (iv) The fate and release mechanisms of particles from face masks are currently unknown, e.g. particles could be released as single particles, as agglomerates, as pieces of fibers containing agglomerates or a combination thereof. Agglomerates are sensitive to changes in the environment such as pH, ionic strength, presence of proteins and motion of the carrier medium, and can de-agglomerate or agglomerate further depending on the environment^30,31^. While this induces complex behavior of nanoparticles in exposure scenarios and in tissue uptake and bio-distribution, influence on toxicity or biological responses remain poorly understood^30,32^. (v) Key information about the toxicity of TiO_2_ particles is missing for risk assessment: data about the hazard (inhalation toxicity threshold) of the specific TiO_2_ particles present in face masks should be determined in a robust, repeated dose inhalation study with fiber-grade TiO_2_ particles. Furthermore, more toxicity and epidemiological research is needed to assess the risk of vulnerable populations, especially children.

## Methods

### ICP-OES sample preparation

The examined face masks consisted of materials that are very resistant to the digestion steps typically applied to prepare samples for total titanium (Ti) analysis by ICP-OES. Adaptation of the sample preparation method based on closed-microwave assisted acid digestion allowed, however, measuring the total amount of Ti. The masks were homogenized by cutting them into small pieces using scissors and mixing the cuts manually. When the masks contained both woven and non-woven textile layers, the layers were digested separately. When the masks contained non-woven textile layers only, making their separation difficult, the entire mask was homogenized.

Two digestion methods were applied, depending on the material. Woven textiles (cotton, polyester or other synthetic fibers) were digested (closed microwave digestion) in a 4:1 (v:v) mixture of nitric acid and sulfuric acid at 220 °C in a Mars 6 microwave (CEM, USA). This method was adapted from the application note for polyethylene terephthalate digestion^33^. The non-woven textiles from the masks needed higher temperatures for complete digestion, and the method was adapted to the light fibers that were not easily wetted. The method uses first a charring step in concentrated sulfuric acid at 260 °C in iPrep vessels (CEM, USA), followed by a digestion step in concentrated nitric acid at 200 °C.

### ICP-OES analysis

After dilution of the digests, the total Ti concentration was determined by ICP-OES at wavelength 368.520 nm (Varian 720, Agilent technologies). All samples were prepared and analyzed in duplicate. Titanium concentrations were recalculated to TiO_2_ concentrations by multiplying them with a factor 1.668, calculated as the ratio of the molecular mass of TiO_2_ (79.88 g/mol) to that of Ti (47.88 g/mol), assuming all Ti is present as TiO_2_.

### TEM sample preparation

A sample preparation methodology for TEM analysis of particles in textiles was developed based on Gashti et al.^34^, Lorenz et al.^35^, Hebeish et al.^36^ and Joshi et al.^37^.

From each mask, a 1 × 1 cm square piece was cut using scissors, and the different layers of the mask were separated. From each layer, a 1 × 5 mm strip was cut. Each strip was transferred into a silicone rubber embedding mold [Silicone Mould 21 Cavity Blue (Agar Scientific Ltd., G3549)] and embedded in EPON812-Spurr resin mixture.

Specimen blocks were trimmed using a TM60 trimming unit (Reichert-Jung A.G., Vienna, Austria) to obtain a cutting face of 0.5–2 mm^2^. Semi-thin sections with a section thickness between 150 and 250 nm were cut using the an Ultracut ultramicrotome (Leica Microsystems, Wetzlar, Germany). The sections were brought on carbon and pioloform-coated 150 mesh copper grids (Agar Scientific Ltd., G2150C; carbon and pioloform layers were added in-house).

### TEM imaging and analysis

Sections of face masks were analyzed using a Talos F200S G2 transmission electron microscope equipped with an HAADF detector and Super-X EDS detector (Thermo Fisher Scientific, Eindhoven, The Netherlands) consisting of 2 windowless silicon drift detectors (SDD) (Thermo Fisher Scientific, Eindhoven, The Netherlands). STEM imaging, aiming to detect, localize and measure the size, morphology and agglomeration state of TiO_2_ particles, and EDX spectra and spectral images, aiming to determine the elemental composition of the observed particles, were recorded using the Velox software (Thermo Fisher Scientific). Descriptive analyses, including elemental analyses, were done in triplicate, based on three individual masks. The size distributions of the constituent particles and of the agglomerates of TiO_2_ particles were estimated by recording ten representative images at high and low magnification, respectively, followed by image analysis using the ImageJ software^38–40^. The magnification selected for quantitative analysis of agglomerates was selected based on the size of the cross sections of the fibers, and was layer dependent. Agglomerate size was determined semi-automatically using the Particlesizer plugin in single particle mode. For quantitative analysis of constituent particles, a magnification of 88,000 times was selected in all cases. Dispersion methods, as applied for the characterization of constituent particles in the E171 food additive^41,42^ and needed to separate the particles from the matrix and for precise subsequent (semi-automatic) image analysis, cannot be applied for the particles in the face masks, which are embedded as agglomerates in a polymer matrix. Therefore, measurement of constituent particles relied on manual measurement of limited datasets, which is relatively imprecise and explains also part of the observed variation in size. The number of measured particles depended on the TiO_2_ concentration in the fibers and ranged from 30 to 166 constituent particles and 12 to 416 agglomerates in ten images at the selected magnification. The raw data resulting from the image analyses was processed using an in-house Python script for calculation of descriptive statistics and for plotting histograms. The surface area of the cross-sections of the fibers was measured based on TEM images using the ImageJ software, and verified by light microscopy.

### Estimation of the fraction of TiO_2_ particles at the fiber surface

The mass of the (agglomerated) TiO_2_ particles at the surface of the fibers in a mask (${M}_{sf}$), can be calculated as:

1 $${M}_{sf}= \mathrm{F} * {M}_{tot}$$

with $\mathrm{F}$ the fraction of the particles at the surface of the fibers and ${M}_{tot}$ the total mass of TiO_2_ in the mask.

Assuming a homogeneous distribution of the agglomerated TiO_2_ particles in the fibers, $\mathrm{F}$ is approximated as the ratio of an external ring-shaped surface of the cross-section of the fibers (S_r_) and the total surface of the cross-section of the fibers (S_cs_) (Supplementary Information 9). The thickness of the external ring-shaped surface, S_r_, is determined by the median diameter (d_a_) of the TiO_2_ agglomerates (Supplementary Information 9). This can be approximated, assuming circular cross-sections of fibers, as:

2 $$\mathrm{F}=\frac{{S}_{r}}{{S}_{CS}}$$

3 $$\mathrm{F}=\frac{\frac{\pi }{4}*{d}_{f}^{2}-\frac{\pi }{4}*({d}_{f}{-{d}_{a})}^{2}}{\frac{\pi }{4}*{d}_{f}^{2}}$$

4 $$\mathrm{F}=\frac{{d}_{f}^{2}-({d}_{f}{-{d}_{a})}^{2}}{{d}_{f}^{2}}$$

with d_f_ the median diameter of the fibers and d_a_ the median minimum Feret diameter of the TiO_2_ agglomerates.

For a specific case of fibers, namely bi-component microfibers, the assumption of a homogeneous distribution of the agglomerated TiO_2_ particles in the fibers is incorrect. Bi-component microfibers are characterized by a larger surface area from which TiO_2_ particles can be released. To account for this increased surface, ${S}_{r, mf}$, a correction factor was introduced:

5 $${S}_{r, mf}={S}_{r}* \frac{\sum \, wedges \, perimeter}{fiber \, perimeter}$$

With $\sum wedges\, perimeter$ the sum of the perimeters of the wedge-shaped (TiO_2_ containing) polyester parts of the fiber, and $fiber\, perimeter$ the perimeter of the (near-)circular cross-section of the microfiber.

## Supplementary Information

Supplementary Information.

## Data Availability

The datasets generated during and/or analysed during the current study are available from the corresponding author on reasonable request.
