# Supplementary material for: Titanium dioxide particles frequently present in face masks intended for general use require regulatory control
Source: Sci Rep. 2022 Feb 15;12:2529. doi: 10.1038/s41598-022-06605-w (PMC8847427; doi:10.1038/s41598-022-06605-w)

## Supplementary Information

# Titanium dioxide particles frequently present in face masks intended for general use require regulatory control

Eveline Verleysen[1], Marina Ledecq[1], Lisa Siciliani[1], Karlien Cheyns[2], Christiane Vleminckx[3], Marie-Noelle Blaude[3], Sandra De Vos[1], Frédéric Brassinne[1], Frederic Van Steen[1], Régis Nkenda[2], Ronny Machiels[2], Nadia Waegeneers[2,3], Joris Van Loco[1,2,3] and Jan Mast[1,2*]

[1] *Trace elements and nanomaterials, Sciensano, Groeselenbergstraat 99, 1180 Uccle, Belgium*
[2] *Trace elements and nanomaterials, Sciensano, Leuvensesteenweg 17, 3080 Tervuren, Belgium*
[3] *Service Risk and Health Impact Assessment, Sciensano, Juliette Wytsmanstraat 14, 1050 Brussels, Belgium*
*corresponding author

**Supplementary Information 1: Images of the examined face masks.**

Mask01

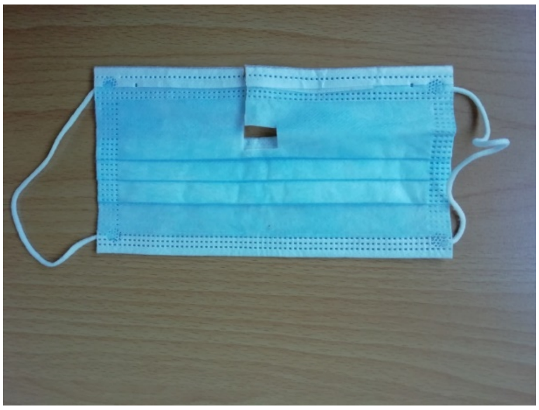

Mask02

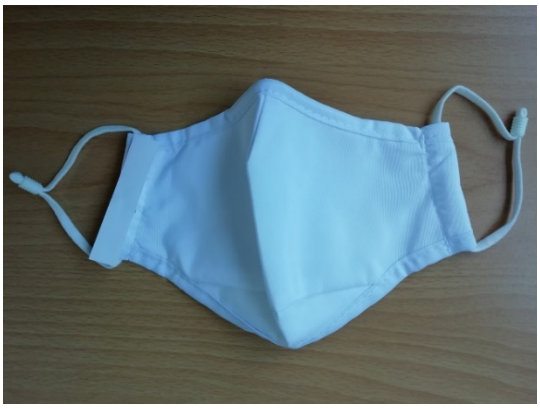

Mask03

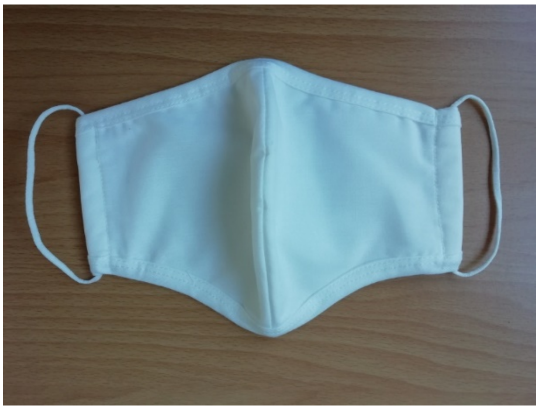

Mask04

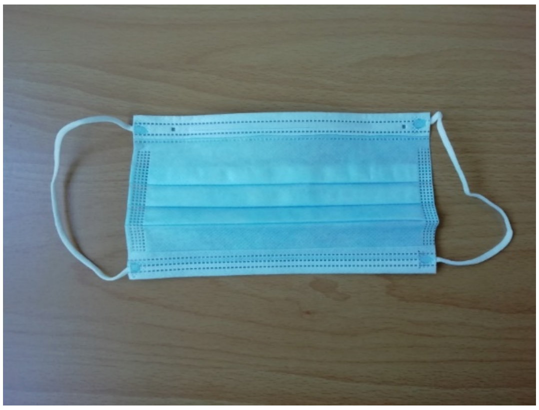

Mask05

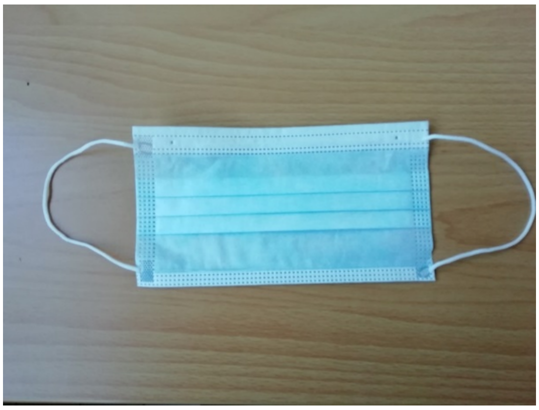

Mask06

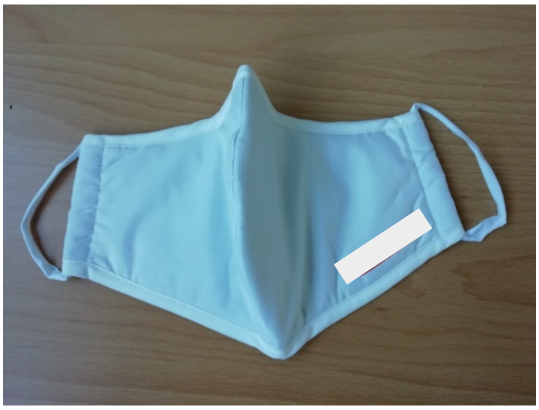

Mask07

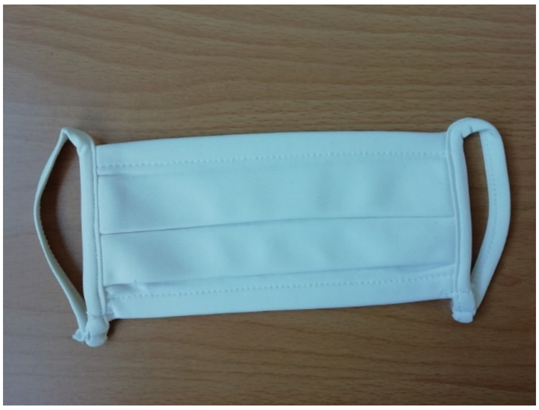

Mask08

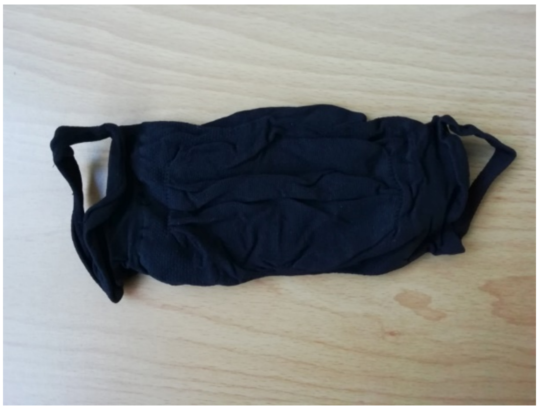

Mask09

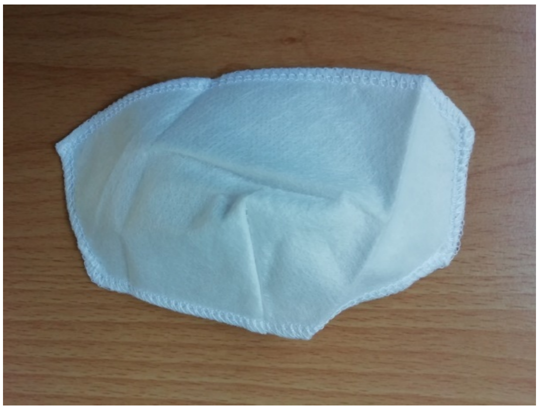

Mask10

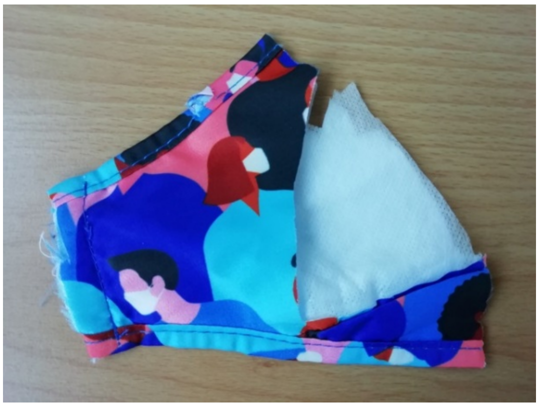

Mask11

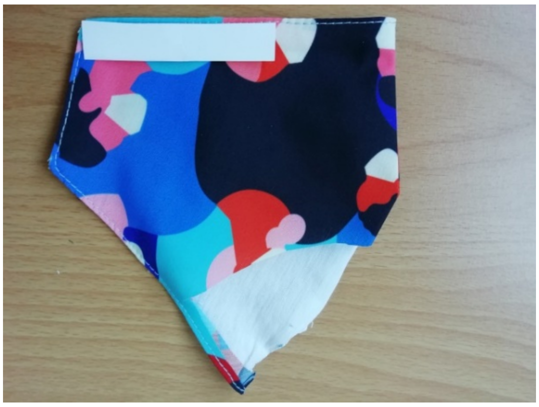

Mask12

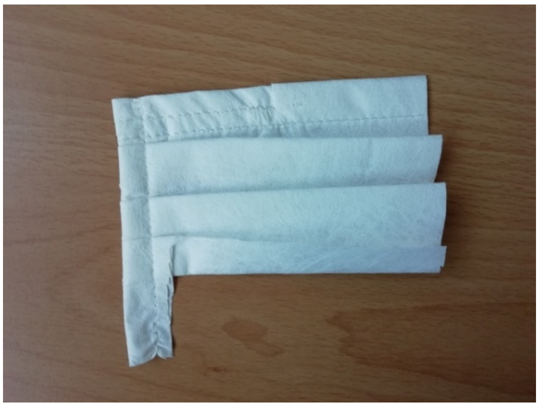

**Supplementary Information 2: Representative HAADF-STEM images of the examined face masks per layer.**

**Mask01: from left to right: external layer, central layer, internal layer**

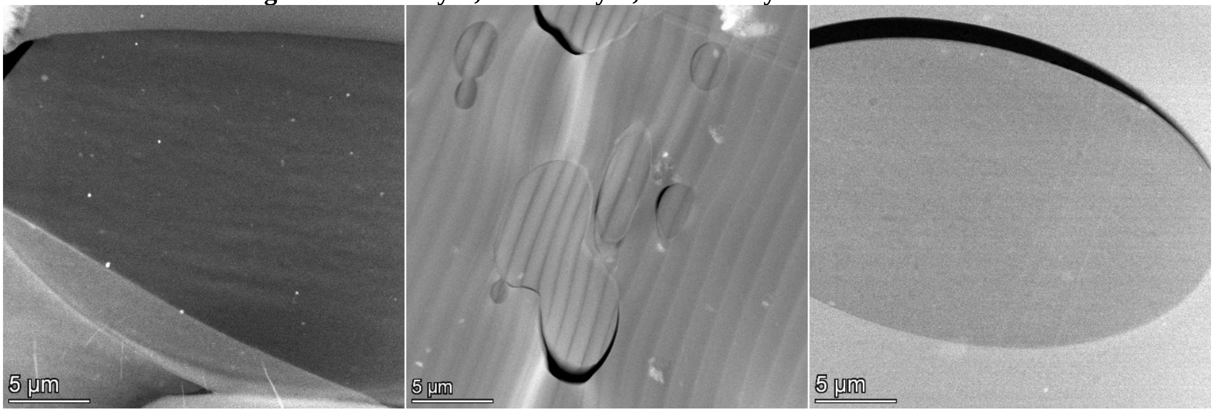


**Mask02: from left to right: external layer, central layer, internal layer**

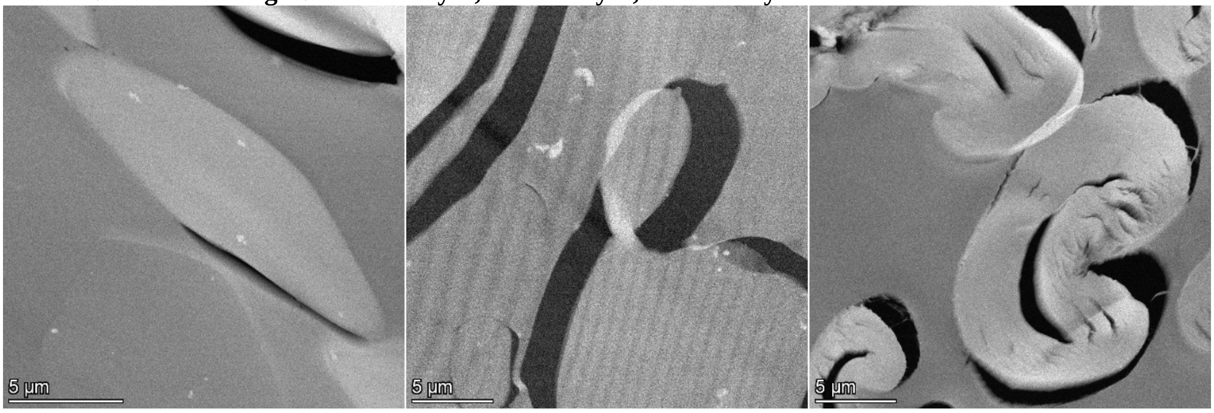


**Mask03: from left to right: external layer, central layer, internal layer**

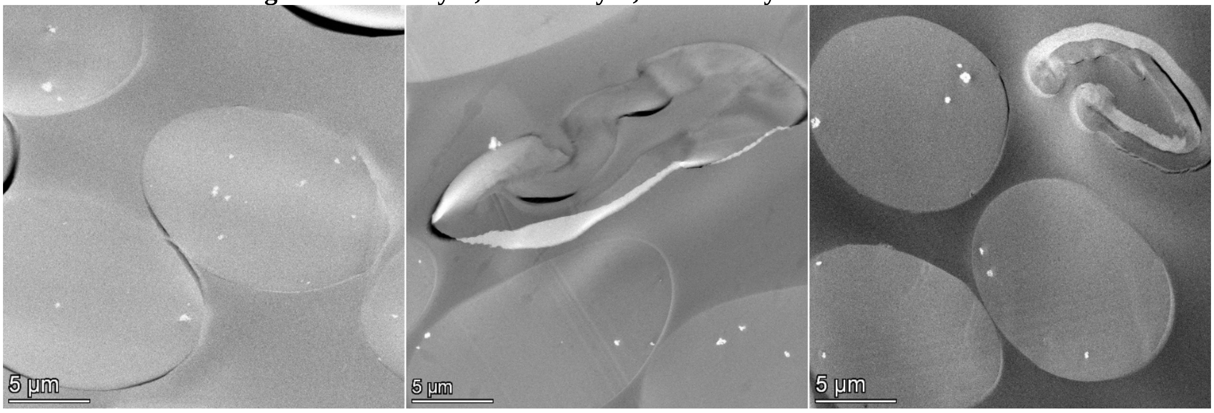


**Mask04: from left to right: external layer, central layer, internal layer**

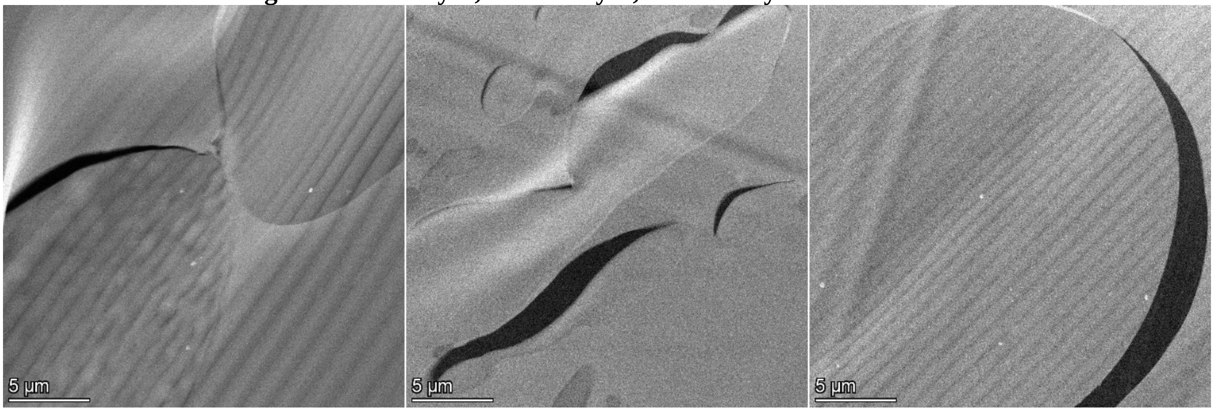

**Mask05: from left to right: external layer, central layer, internal layer**

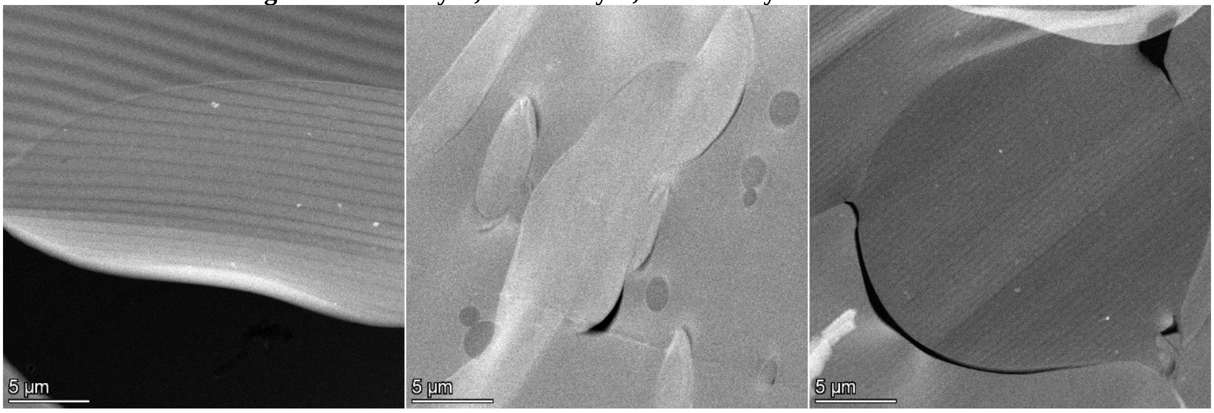


**Mask06: from left to right: external layer, central layer, internal layer**

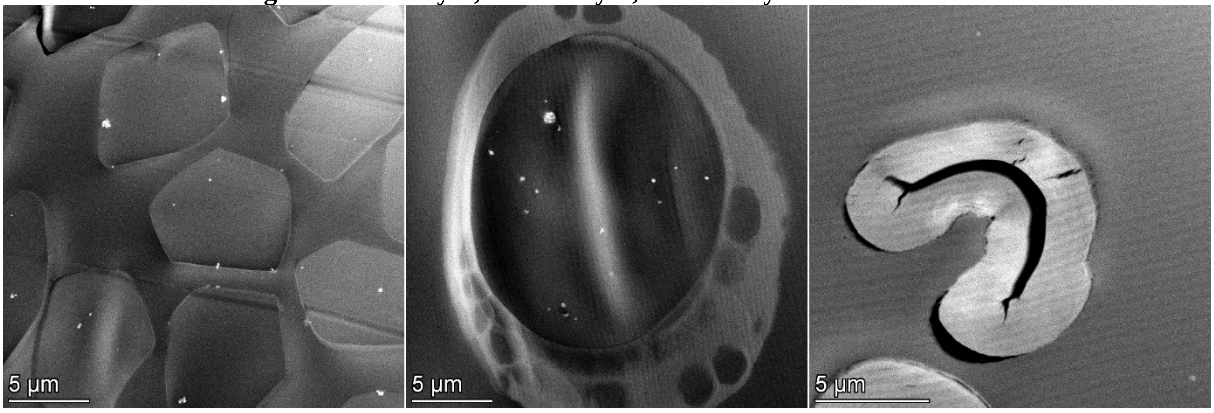


**Mask07: from left to right: external layer, internal layer**

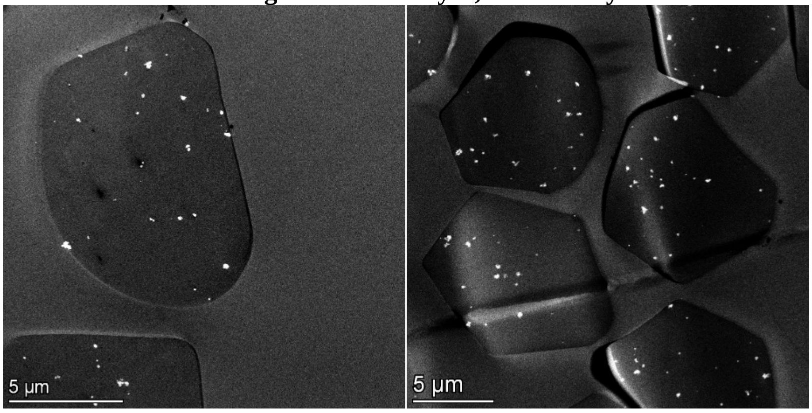


**Mask08**

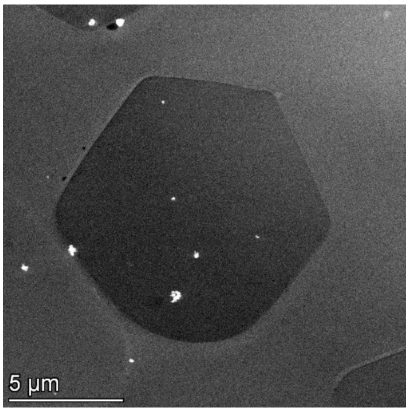

**Mask09: from left to right: external layer, central layer, internal layer**

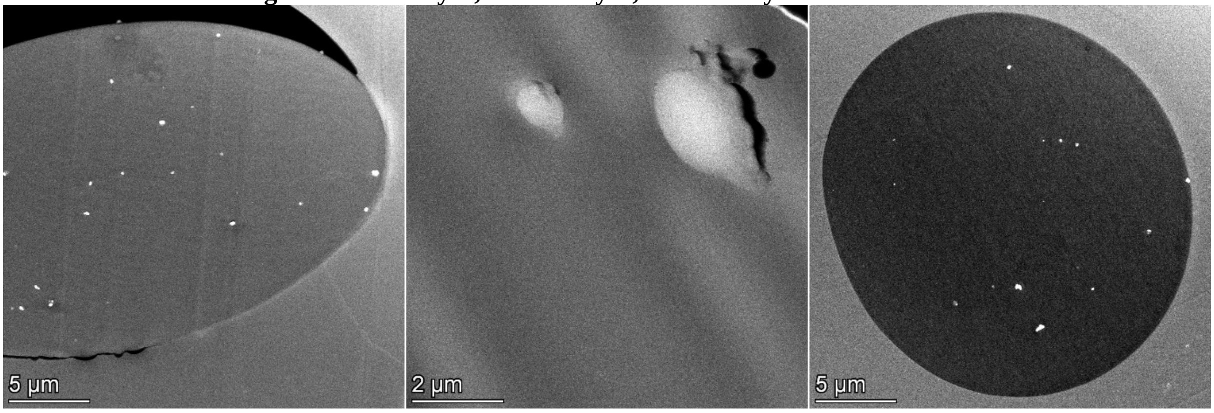


**Mask10: from left to right: external layer, internal layer**

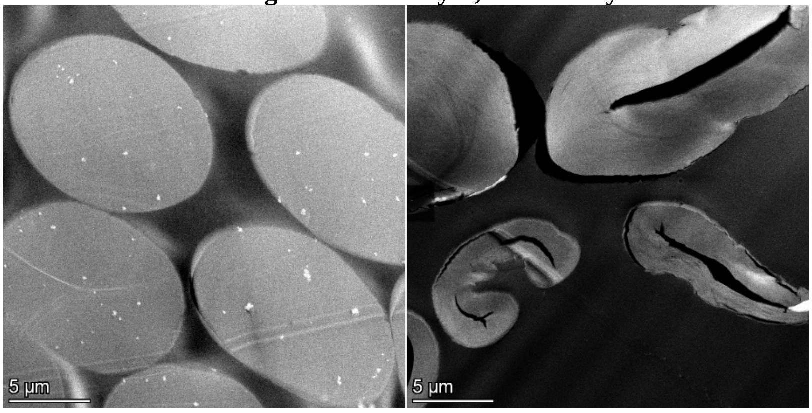


**Mask11: from left to right: external layer, central layer, internal layer**

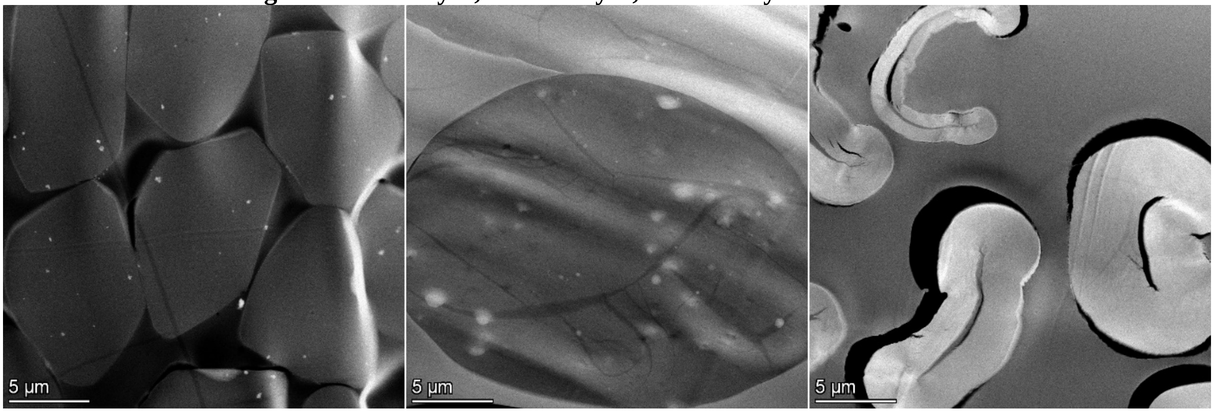


**Mask12: from left to right: external layer, internal layer**

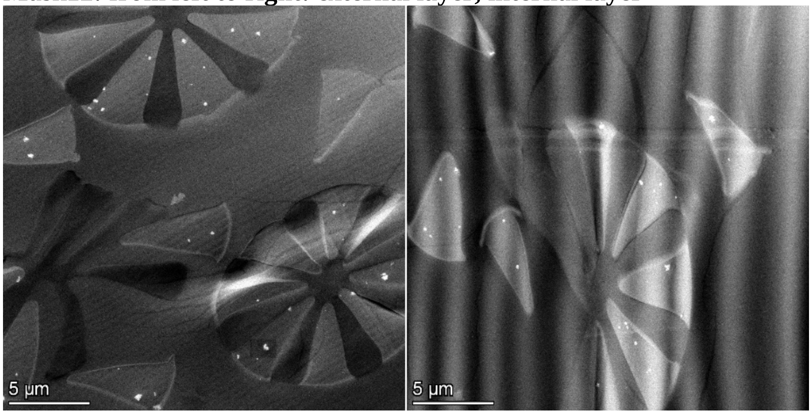

**Supplementary Information 3: STEM-EDX analysis results of all layers containing $TiO_2$.** (first column) The low magnification HAADF-STEM images show the cross sections of the fibers containing the analyzed particles (white arrows) shown in the (second column) higher magnification HAADF-STEM images. (third column) The spectral images of Ti (green) obtained by EDX show that the measured Ti signal coincides with the position of the particles shown in the STEM image, and (fourth column) the EDX spectra of the area's indicated on the STEM image show the Ti signal.

**Mask01, external layer**

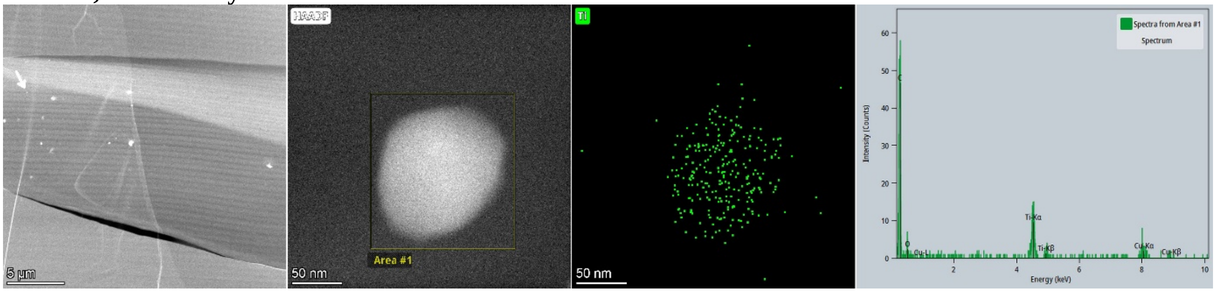


**Mask02, external layer**

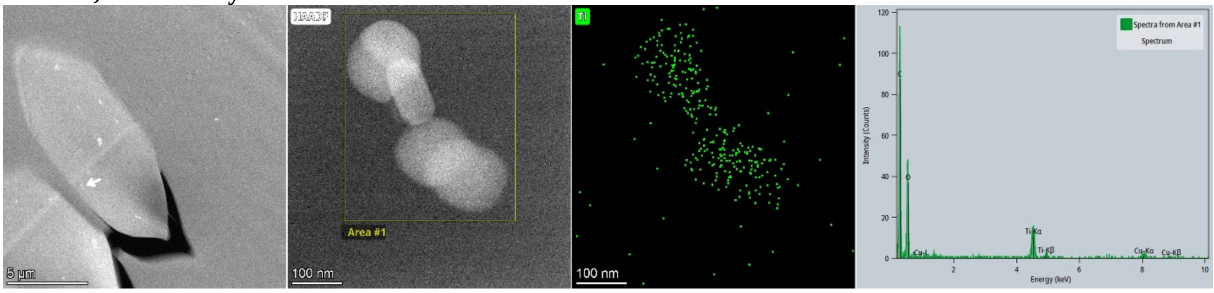


**Mask03, external layer**

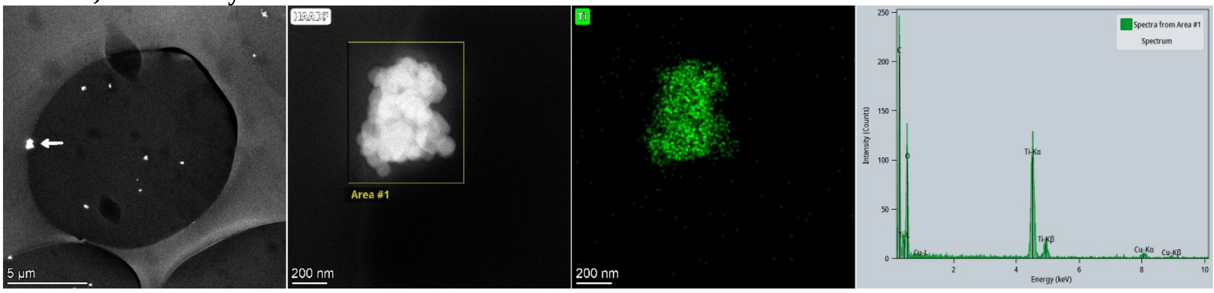


**Mask03, central layer**

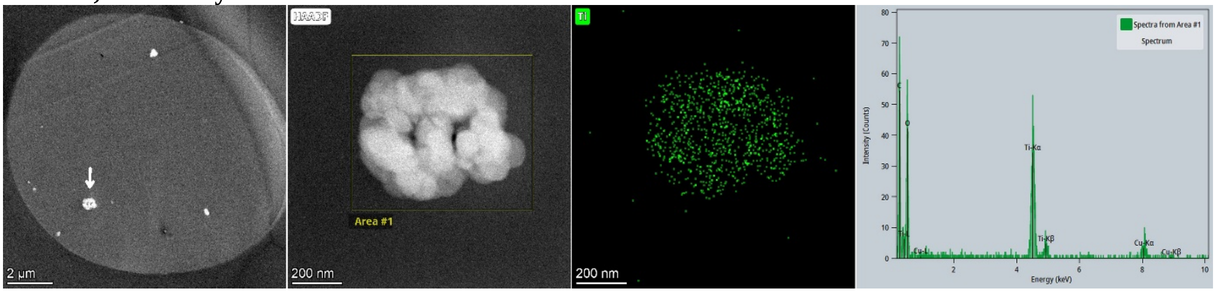


**Mask03, internal layer**

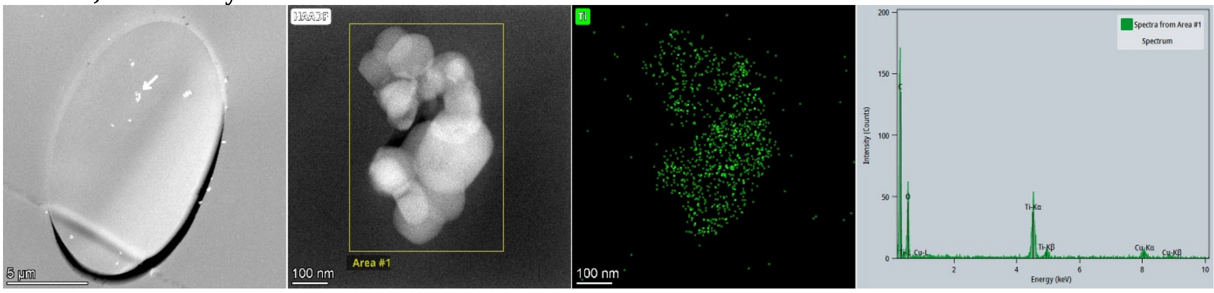

## Mask04, external layer

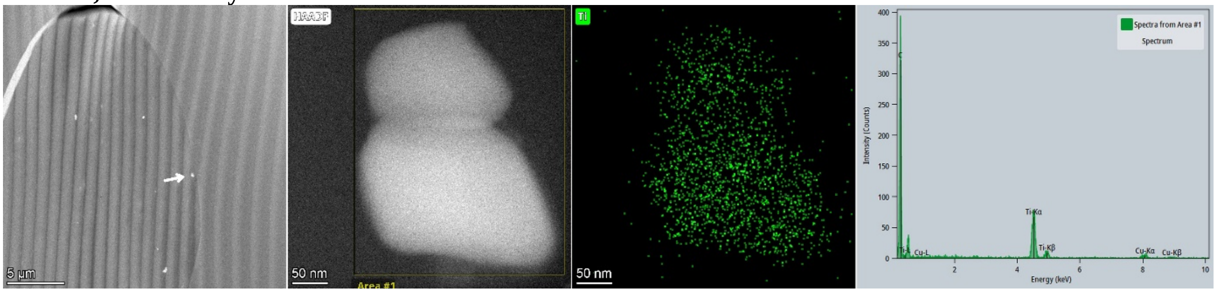


## Mask04, internal layer

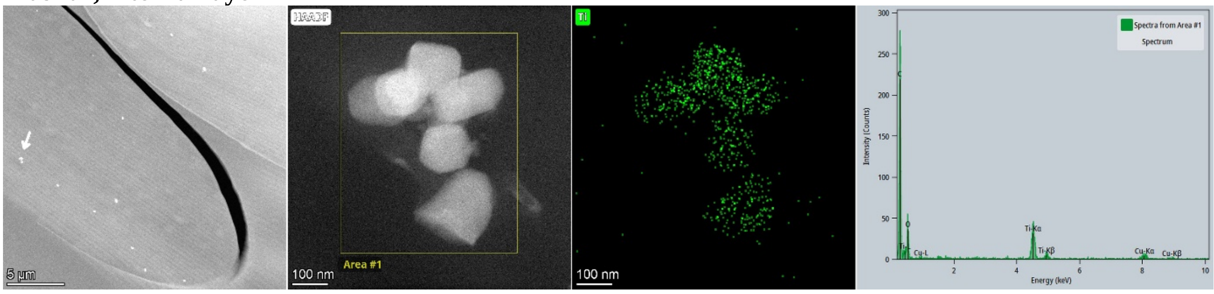


## Mask05, external layer

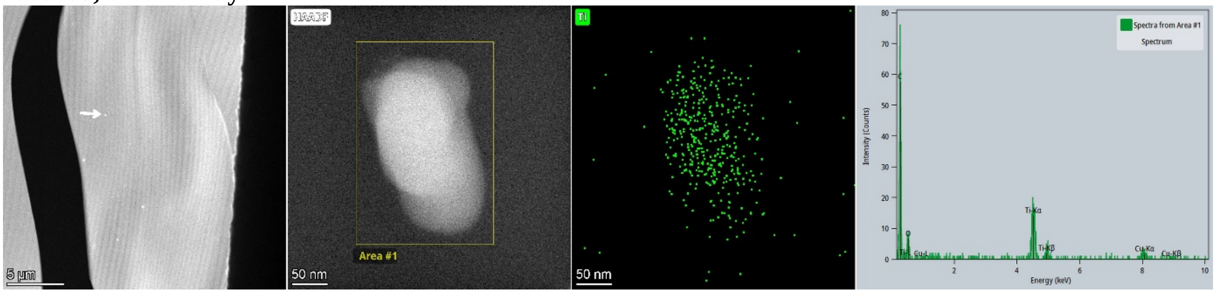


## Mask05, internal layer

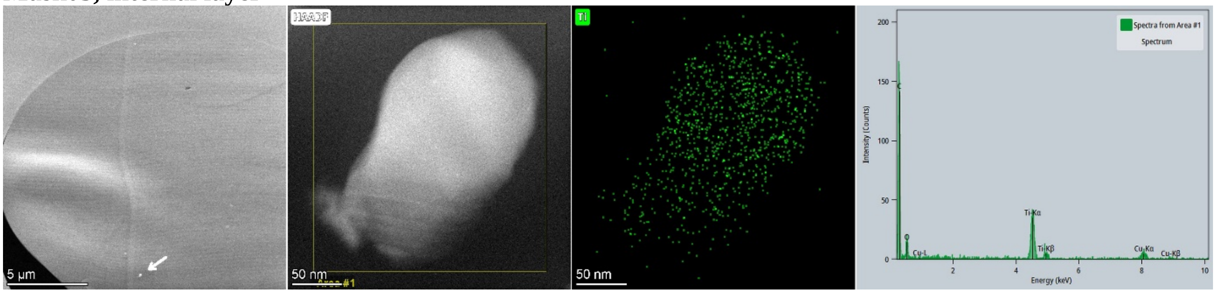


## Mask06, internal layer

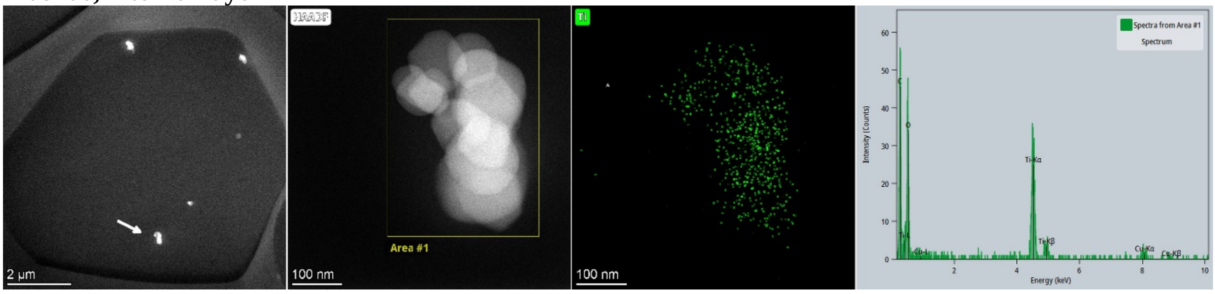

## Mask06, central layer

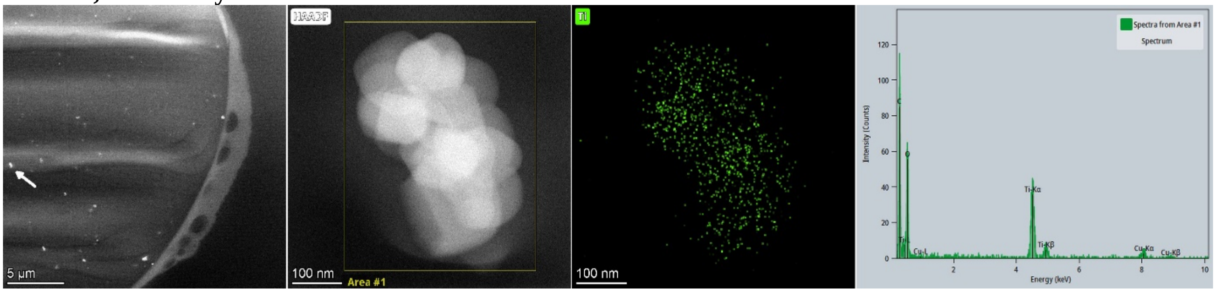


## Mask07, external layer

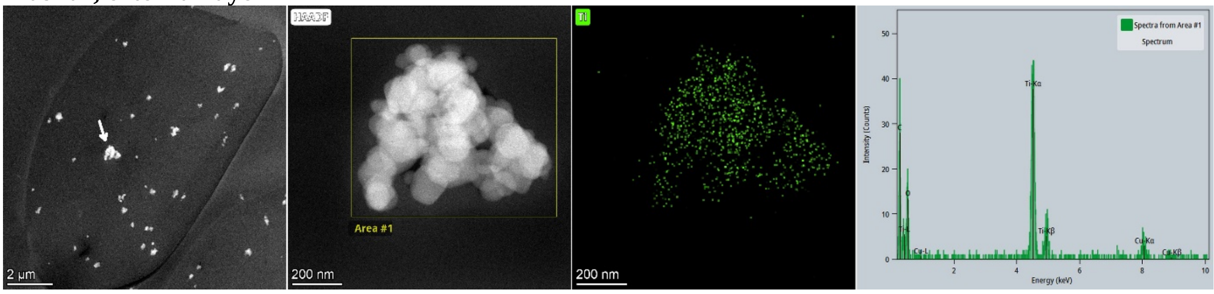


## Mask07, internal layer

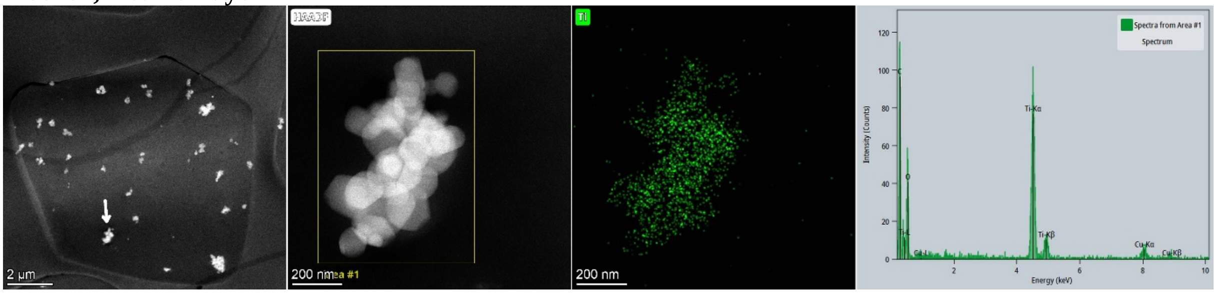


## Mask08

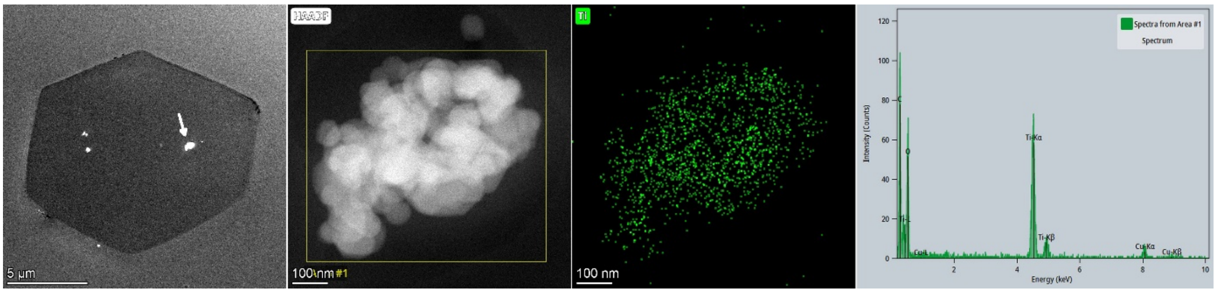


## Mask09, external layer

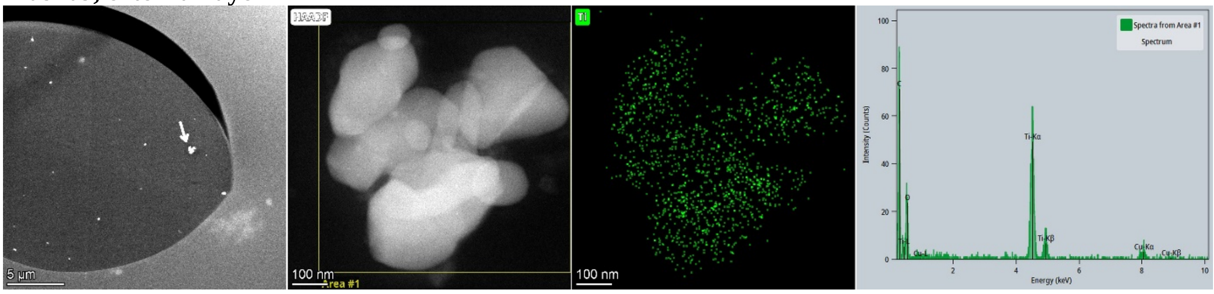

**Mask09, internal layer**

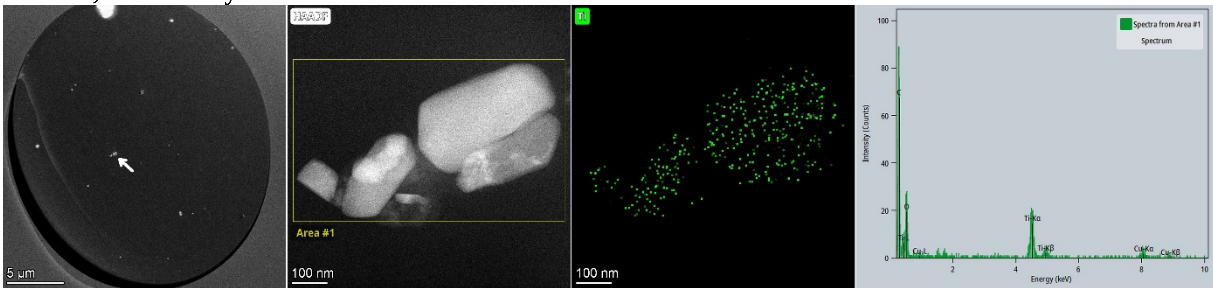


**Mask10, external layer**

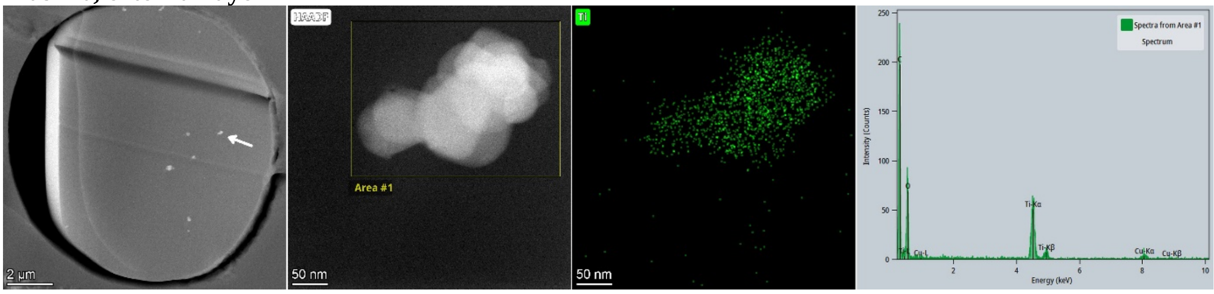


**Mask11, external layer**

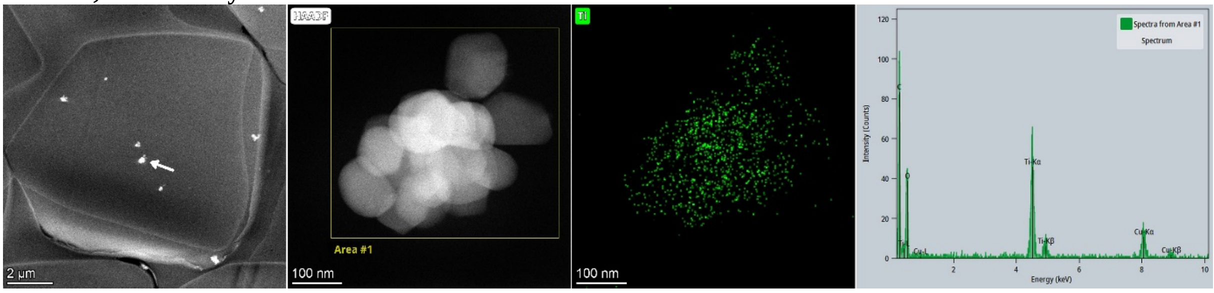


**Mask12, external layer**

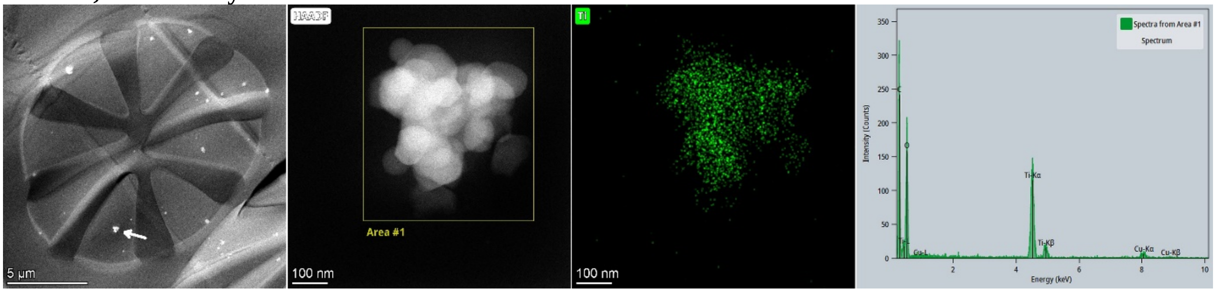


**Mask12, internal layer**

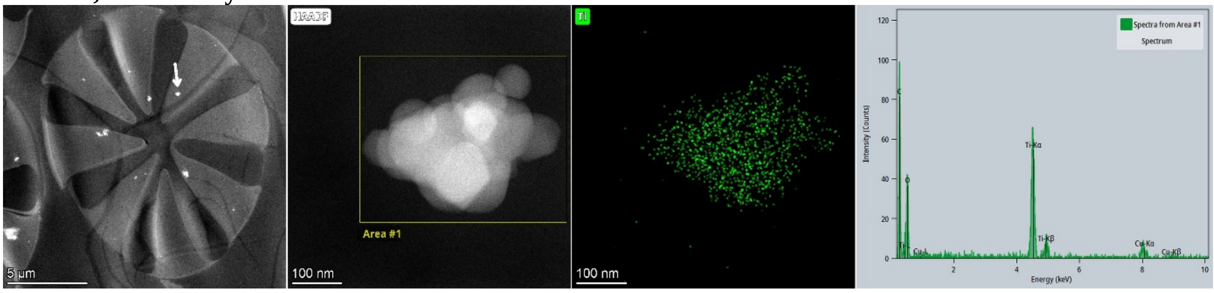

**Supplementary Information 4: Size distributions (minimum Feret diameter, Feret diameter) of $TiO_2$ agglomerates.**

## Mask01, external layer

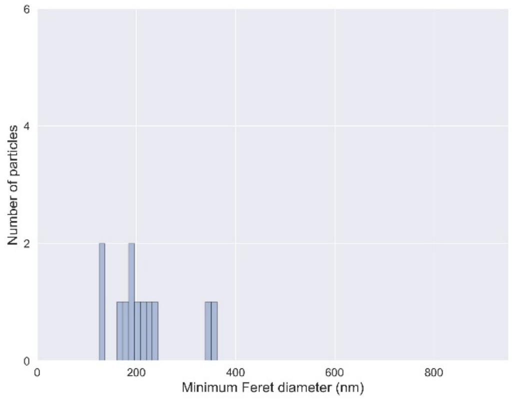


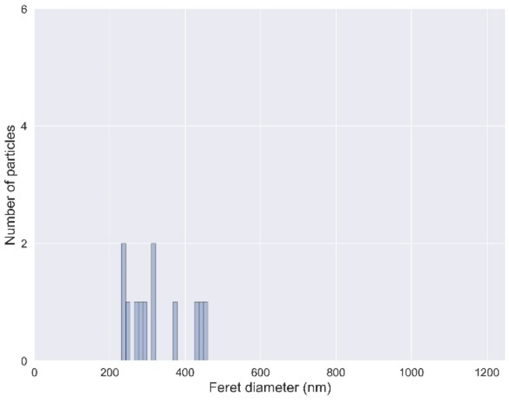


## Mask02, external layer

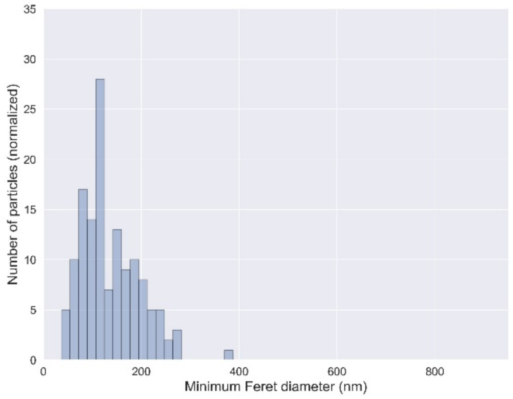


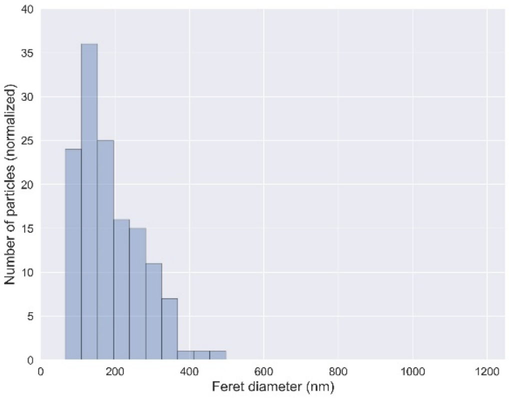


## Mask03, external layer

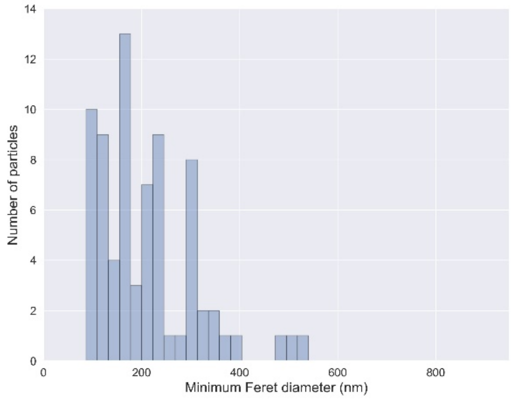


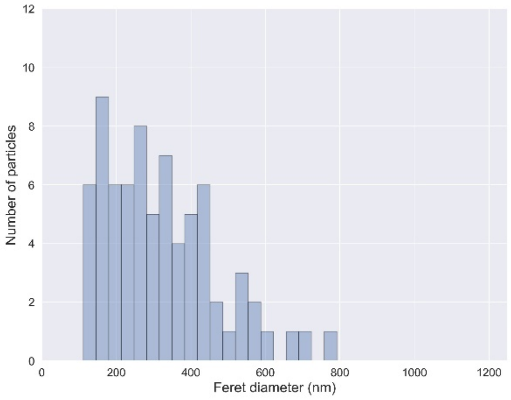


## Mask03, central layer

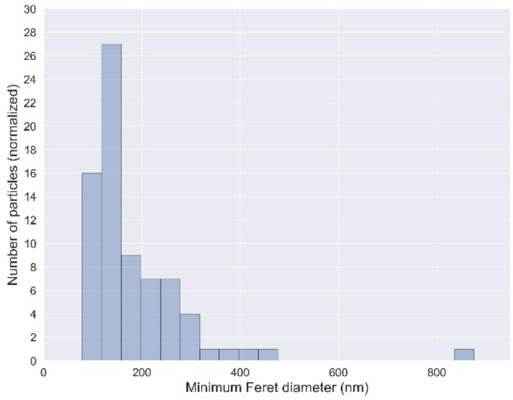


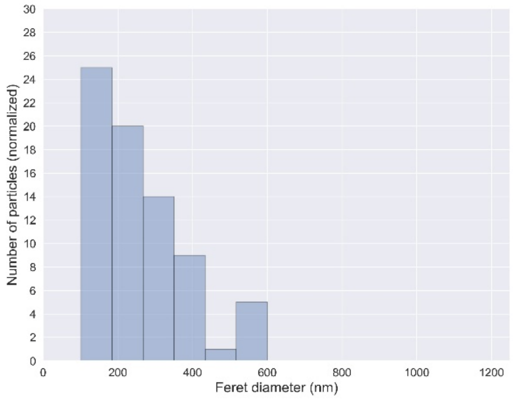

## Mask03, internal layer

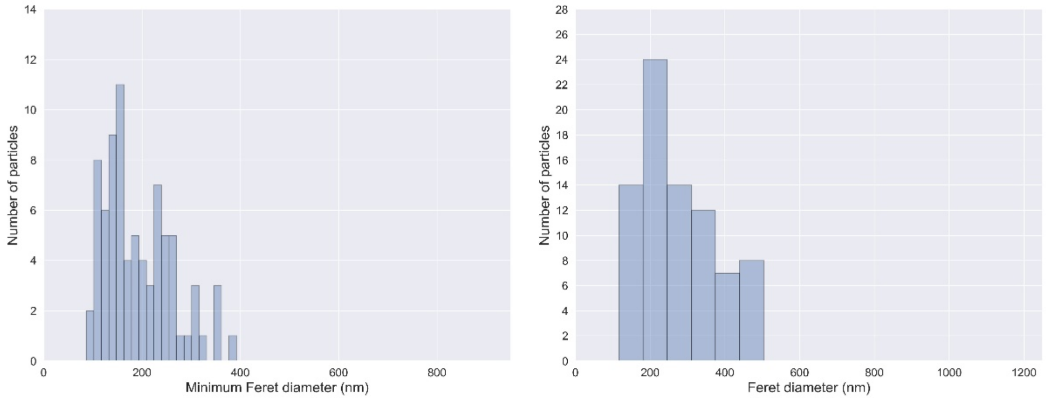


## Mask04, external layer

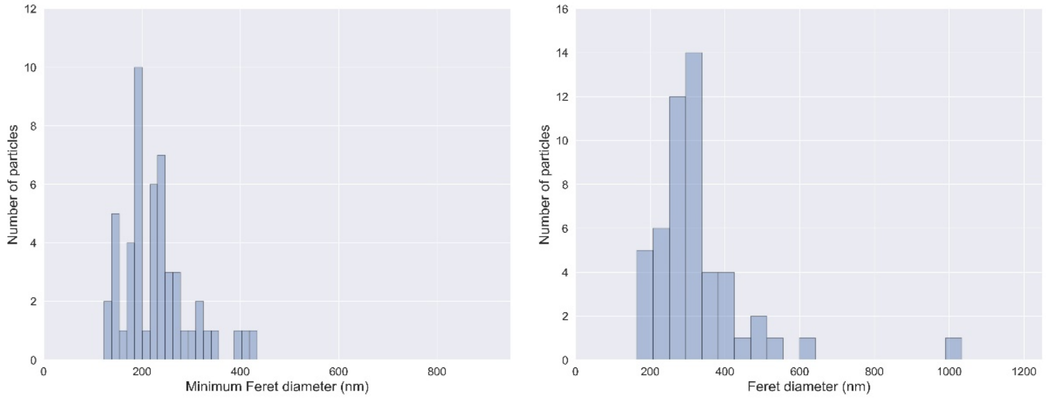


## Mask04, internal layer

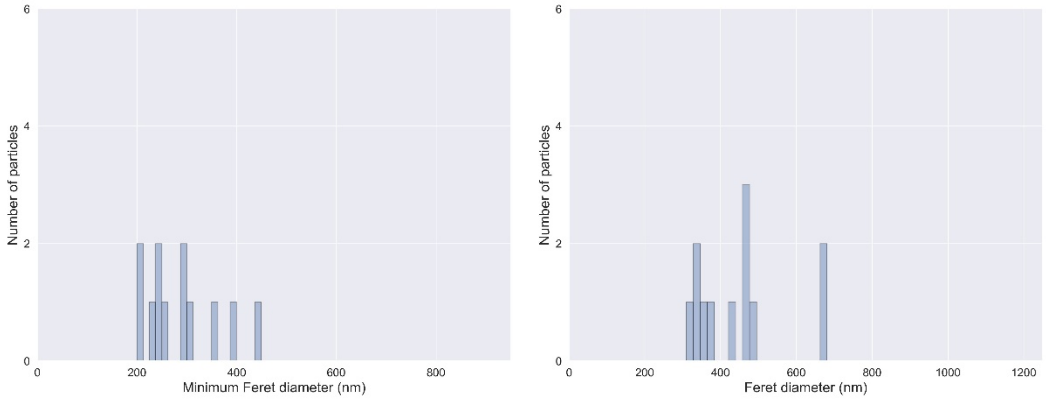


## Mask05, external layer

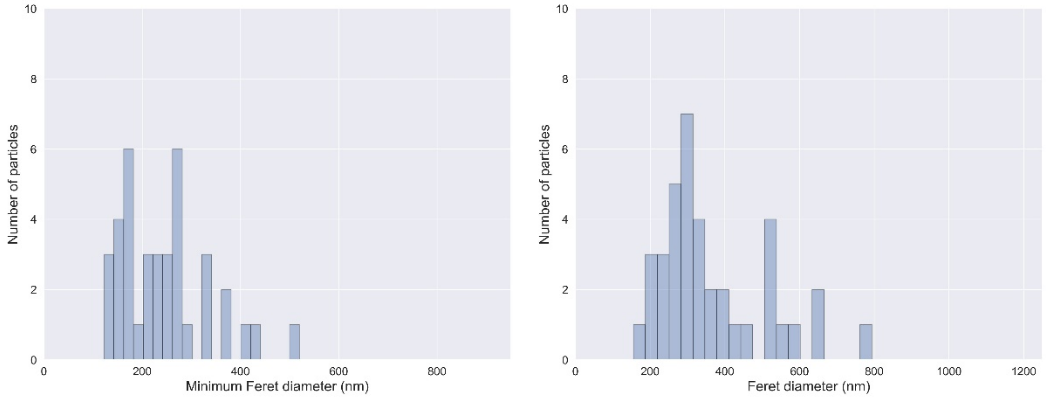

**Mask05, internal layer**

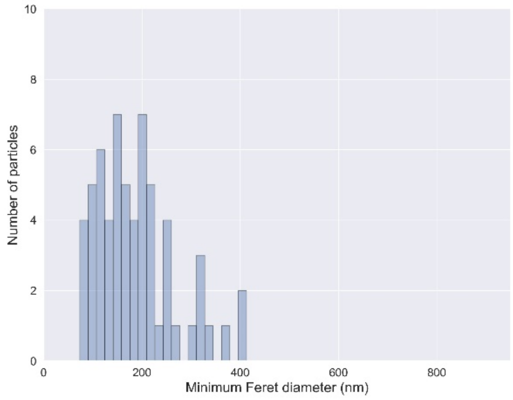


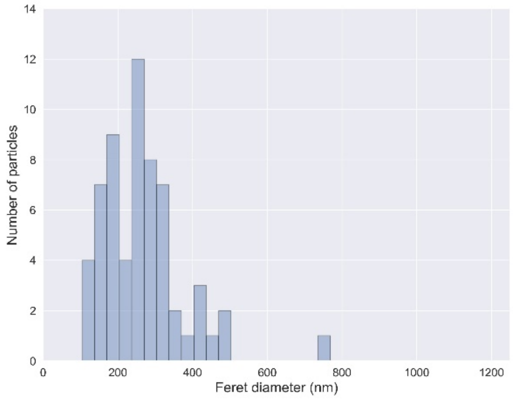


**Mask06, external layer**

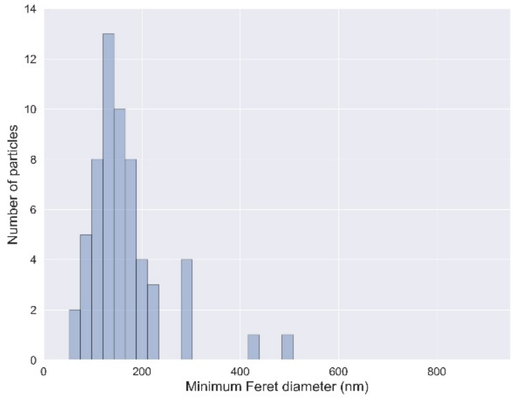


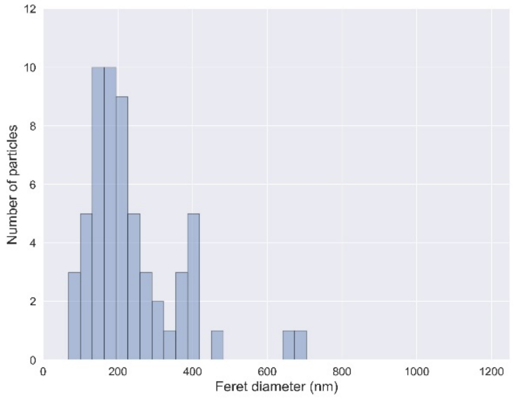


**Mask06, central layer**

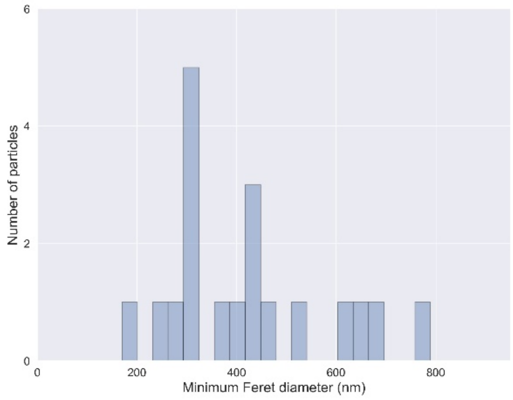


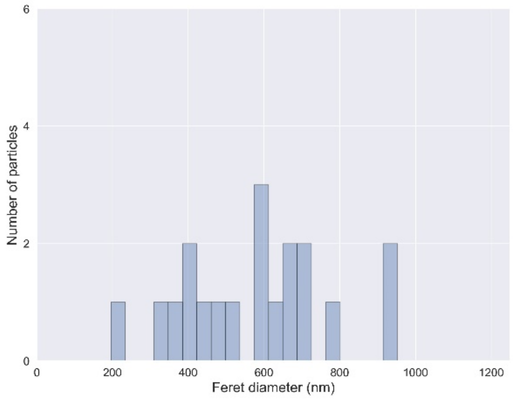


**Mask07, external layer**

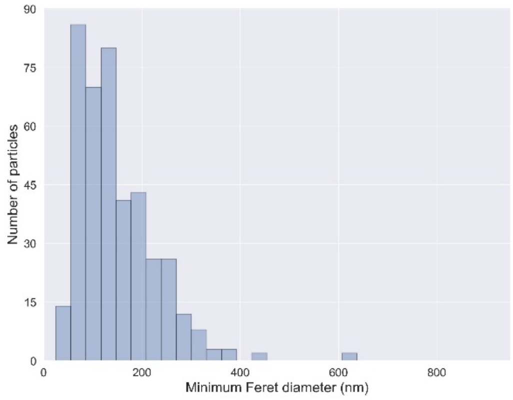


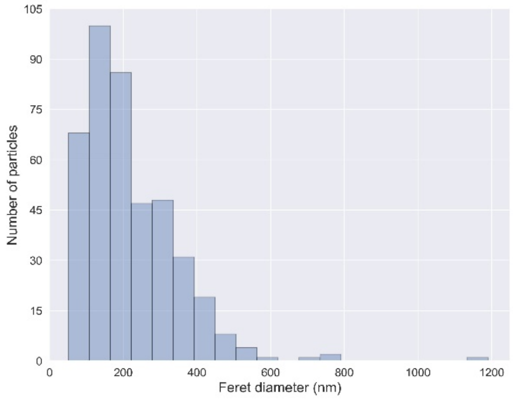

**Mask07, internal layer**

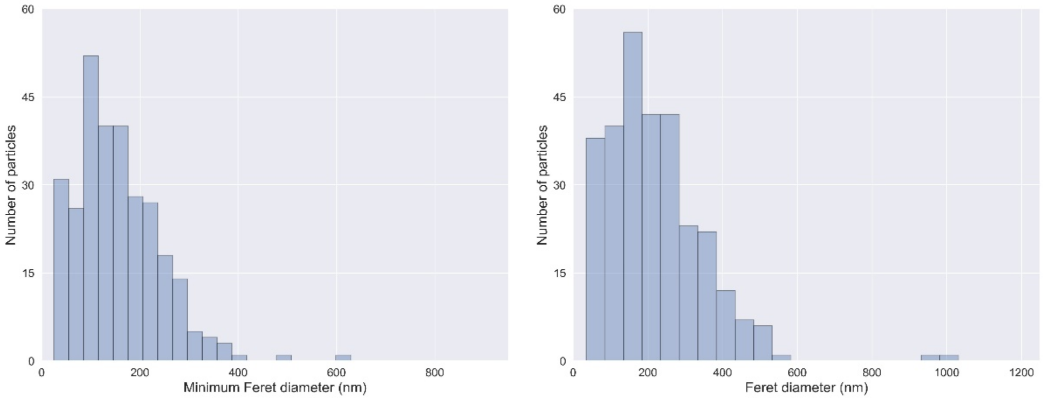


**Mask08**

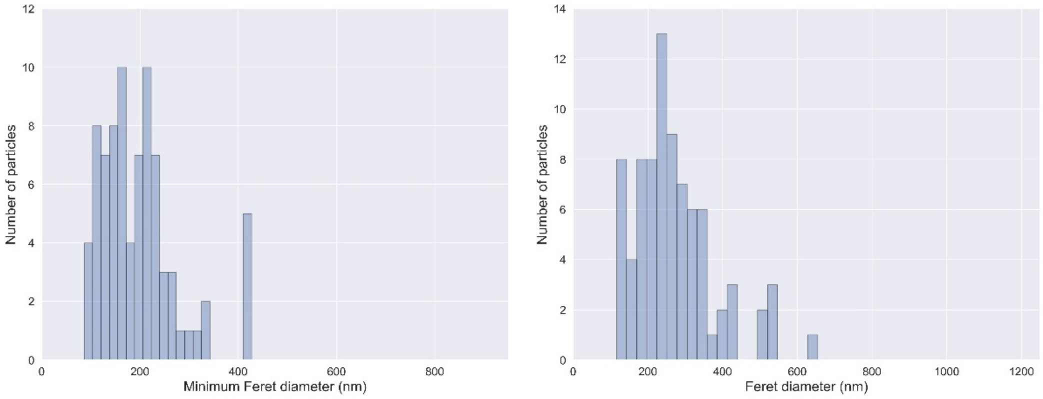


**Mask09, external layer**

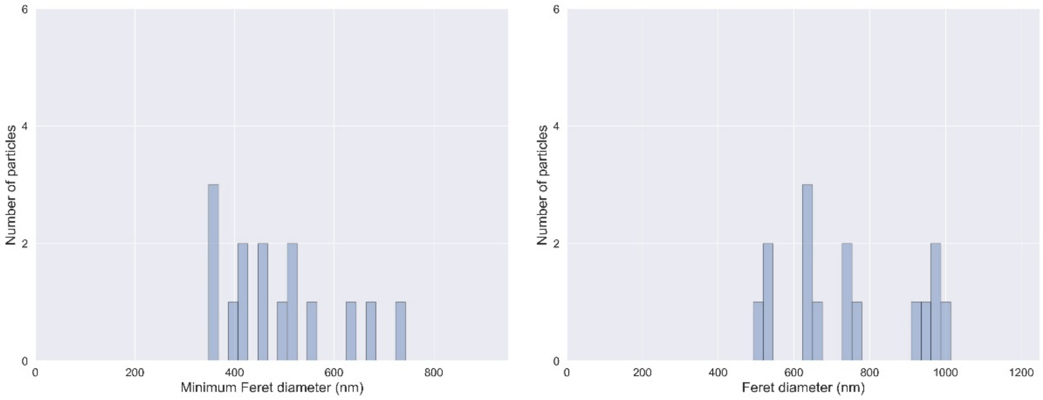


**Mask09, internal layer**

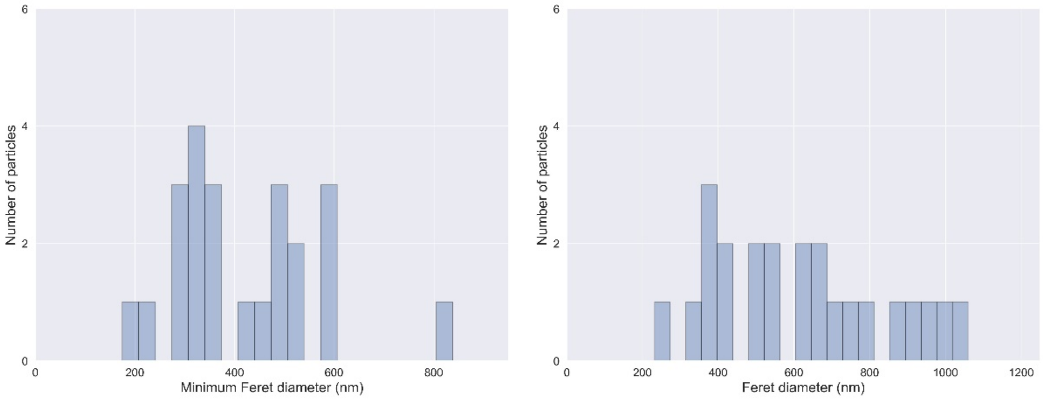

**Mask10, external layer**

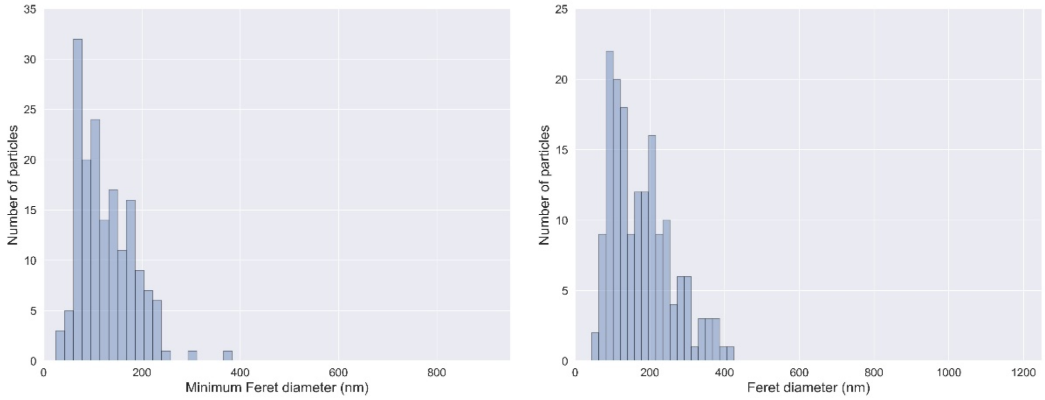


**Mask11, external layer**

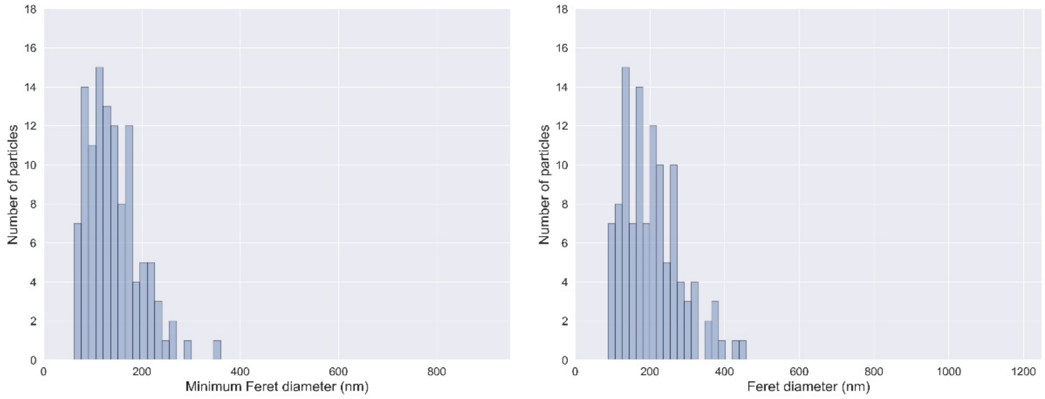


**Mask12, external layer**

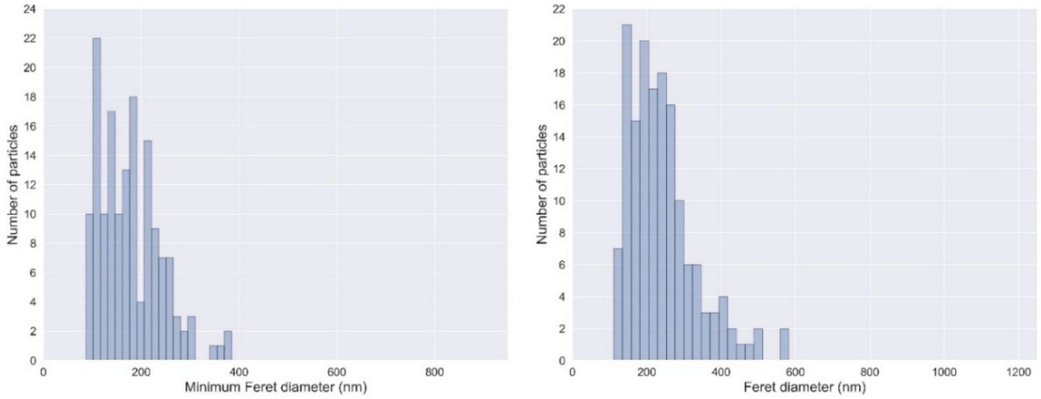


**Mask12, internal layer**

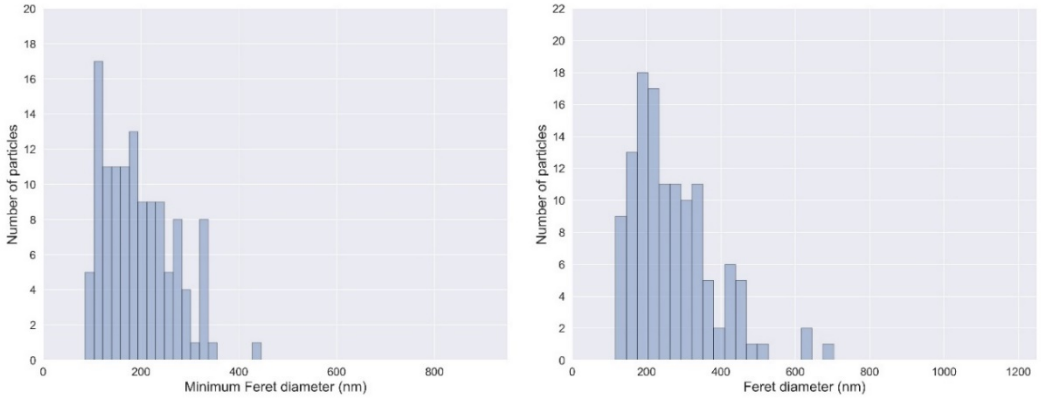

**Supplementary Information 5: Size distributions (minimum Feret diameter, Feret diameter) of $TiO_2$ constituent particles.**

### Mask01, external layer

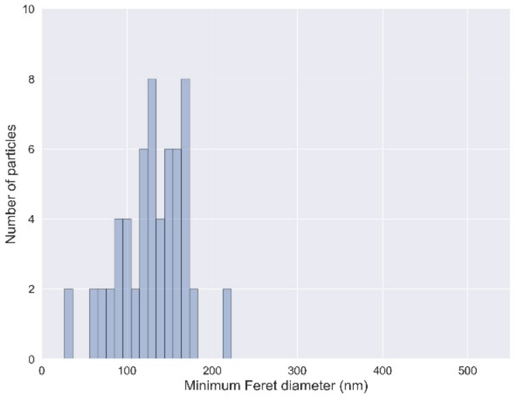


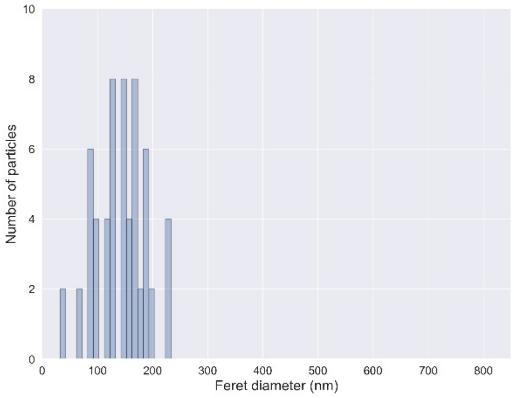


### Mask02, external layer

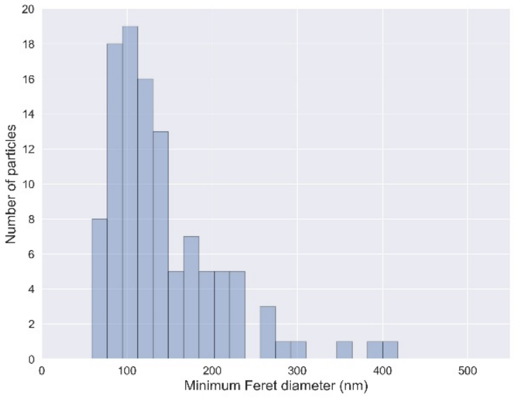


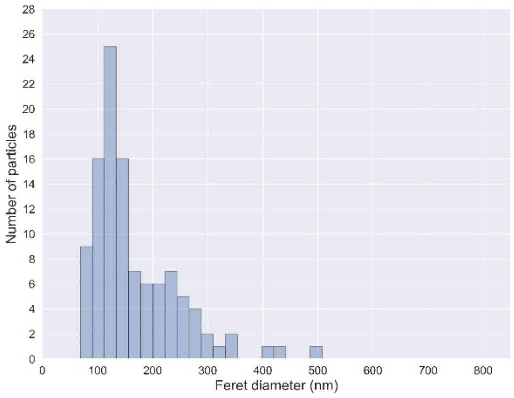


### Mask03, external layer

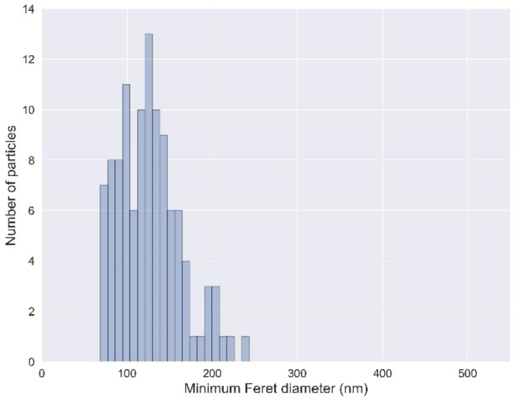


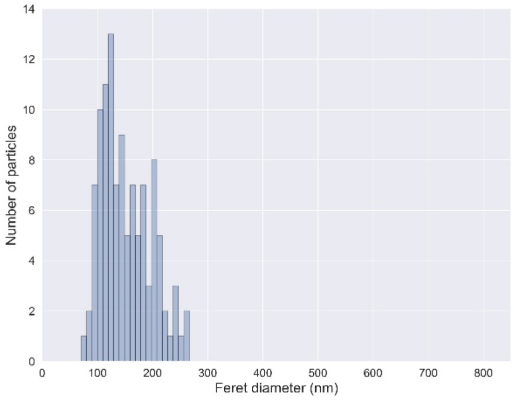


### Mask03, central layer

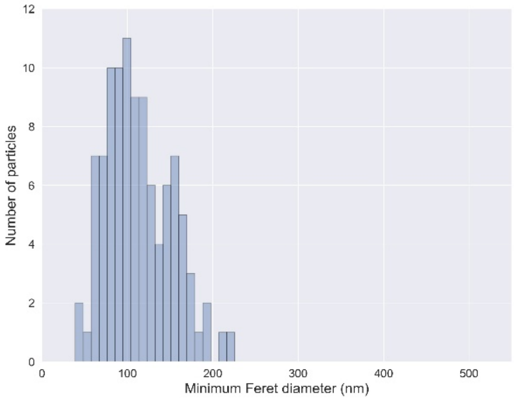


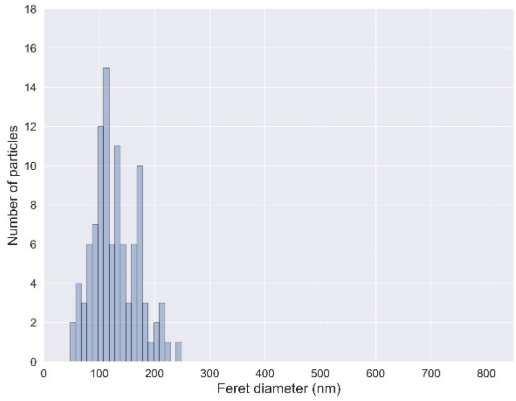

**Mask03, internal layer**

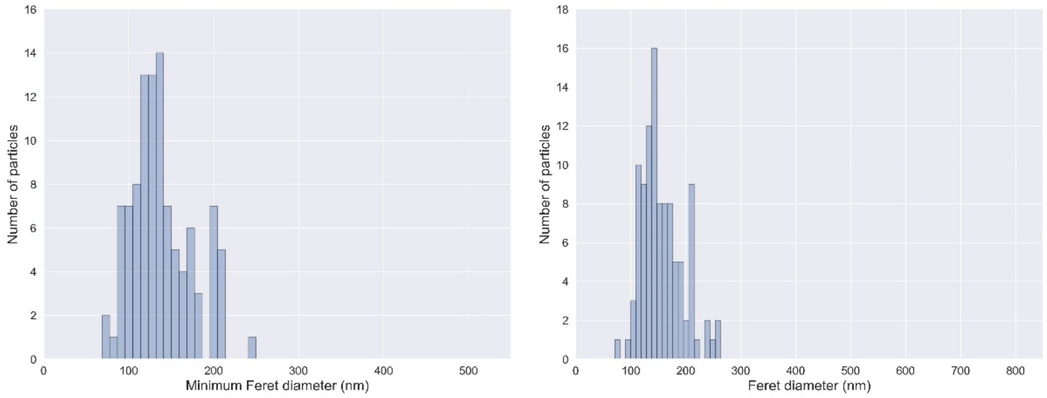


**Mask04, external layer**

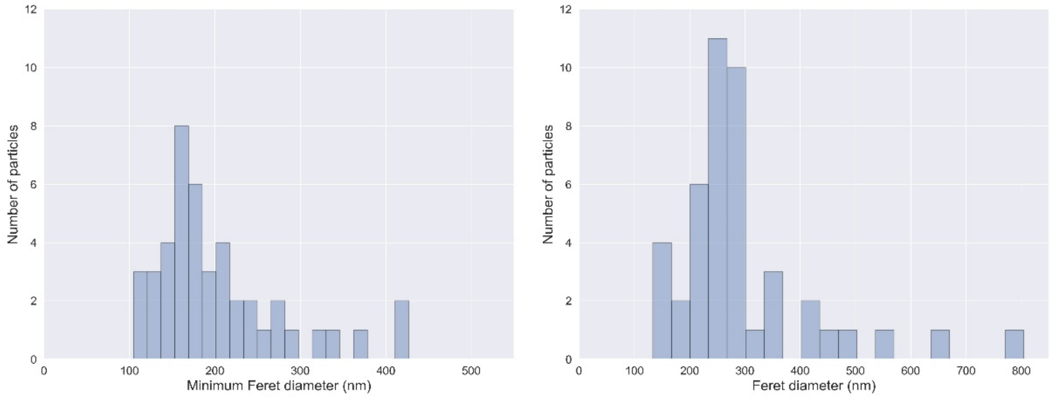


**Mask04, internal layer**

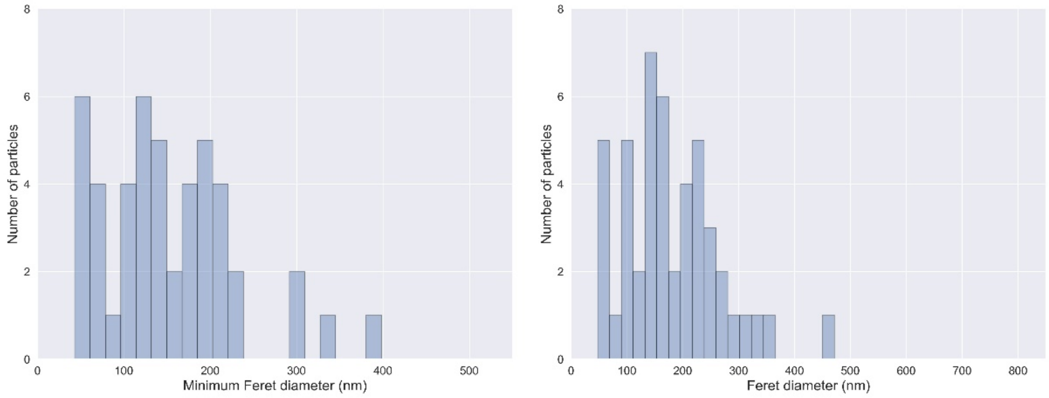


**Mask05, external layer**

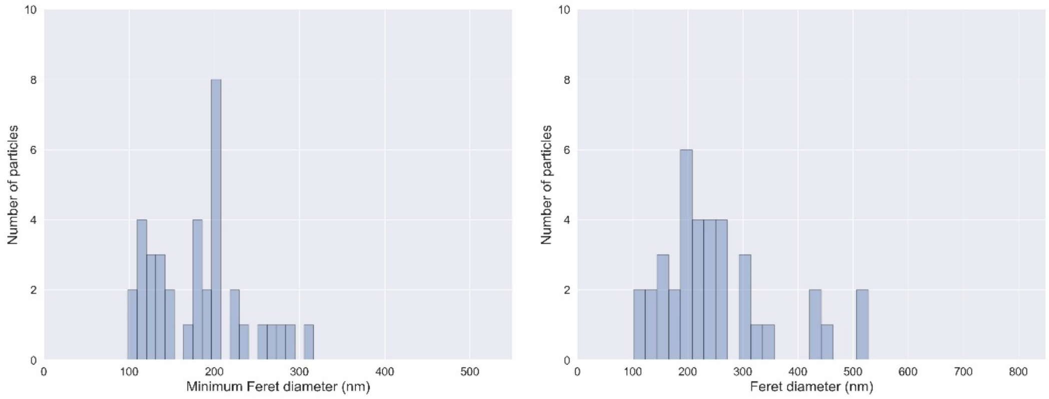

## Mask05, internal layer

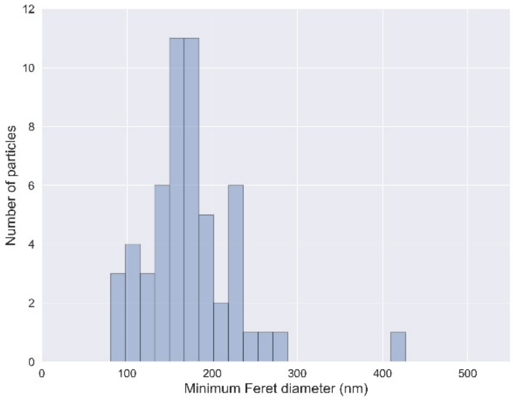


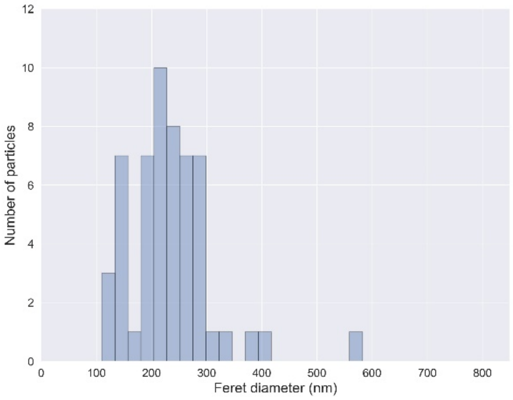


## Mask06, external layer

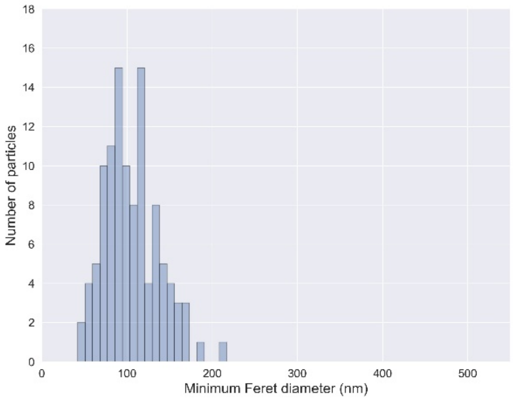


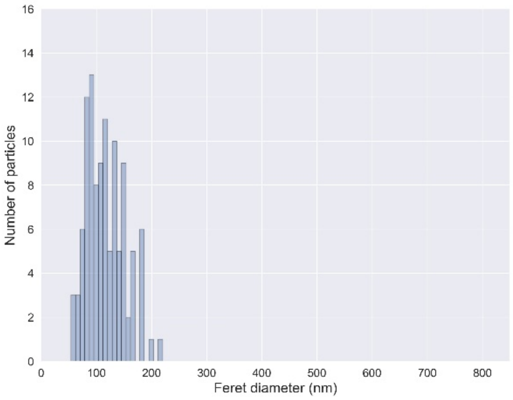


## Mask06, central layer

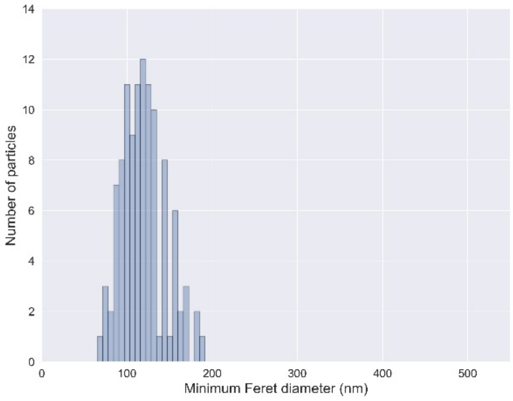


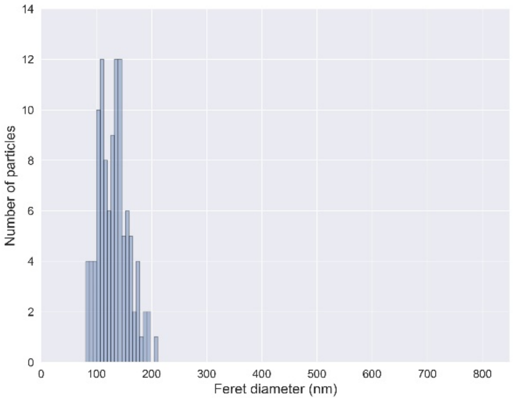


## Mask07, external layer

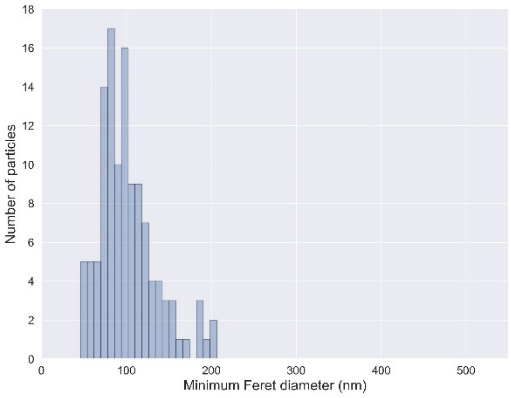


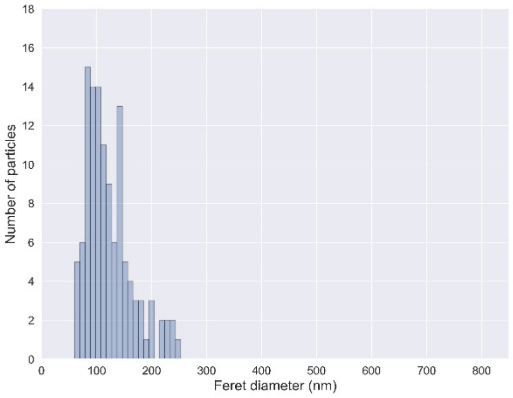

## Mask07, internal layer

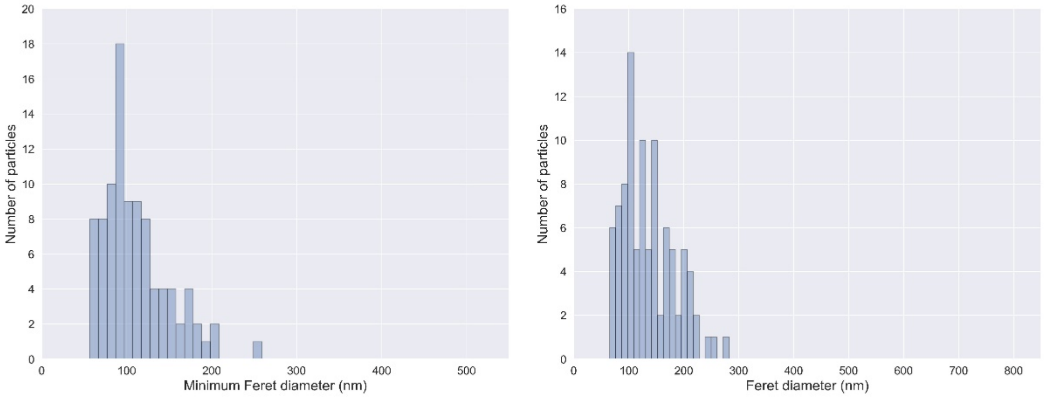


## Mask08

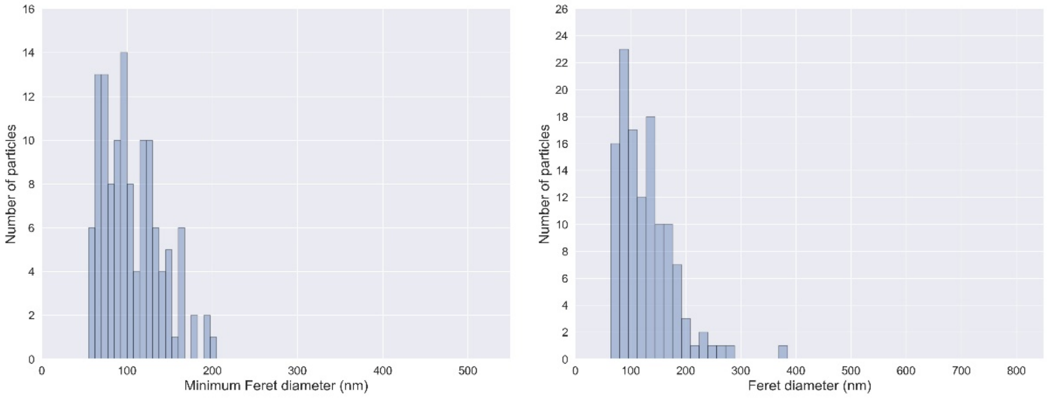


## Mask09, external layer

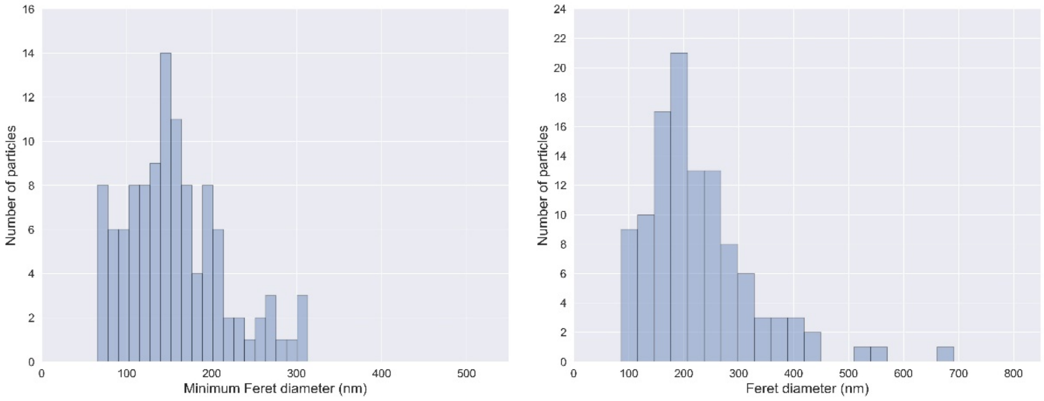


## Mask09, internal layer

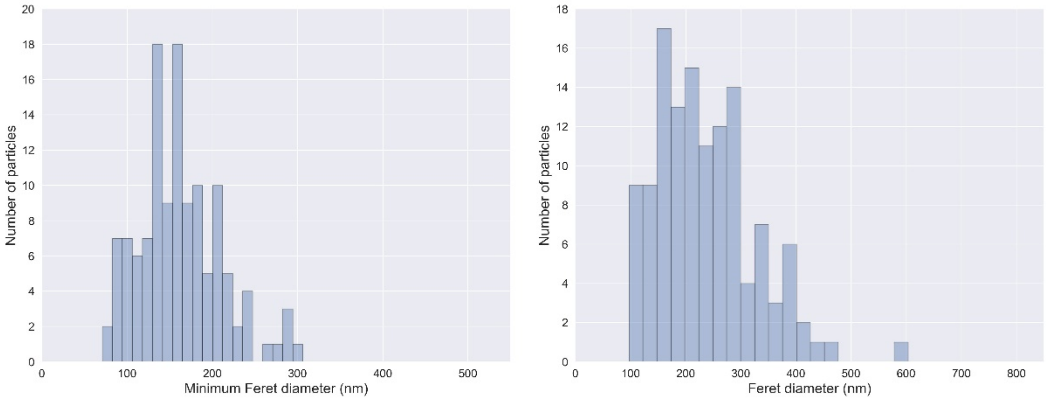

**Mask10, external layer**

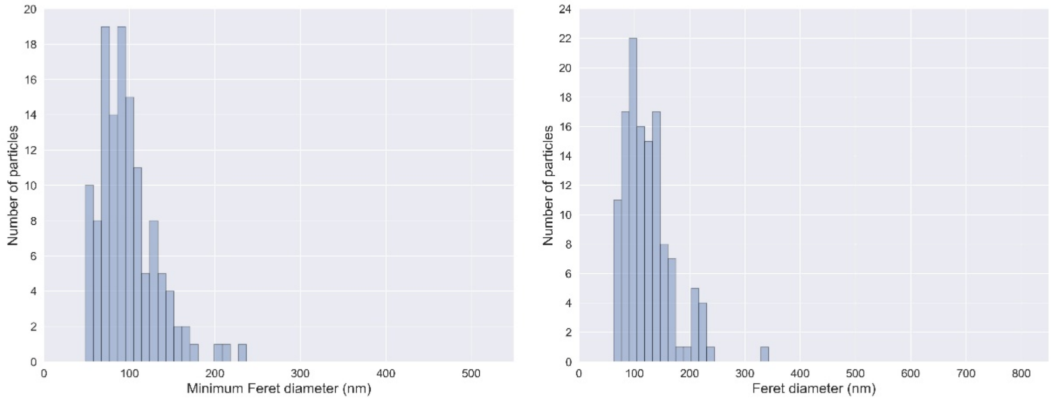


**Mask11, external layer**

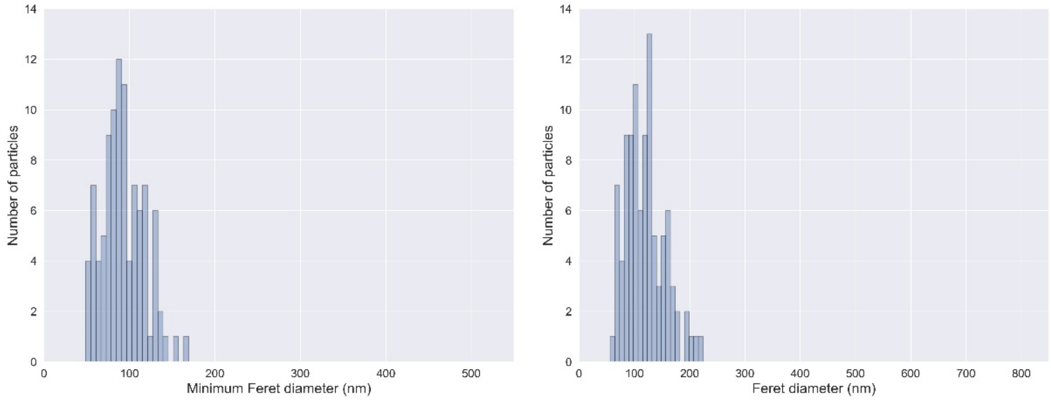


**Mask12, external layer**

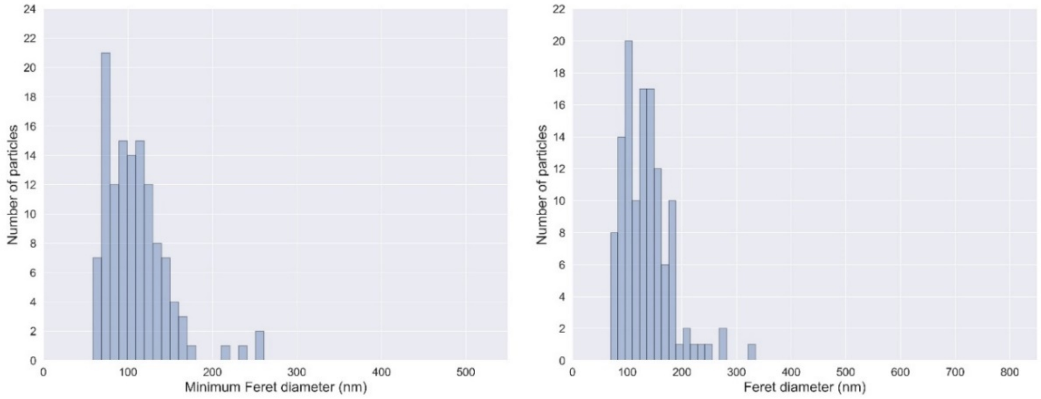


**Mask12, internal layer**

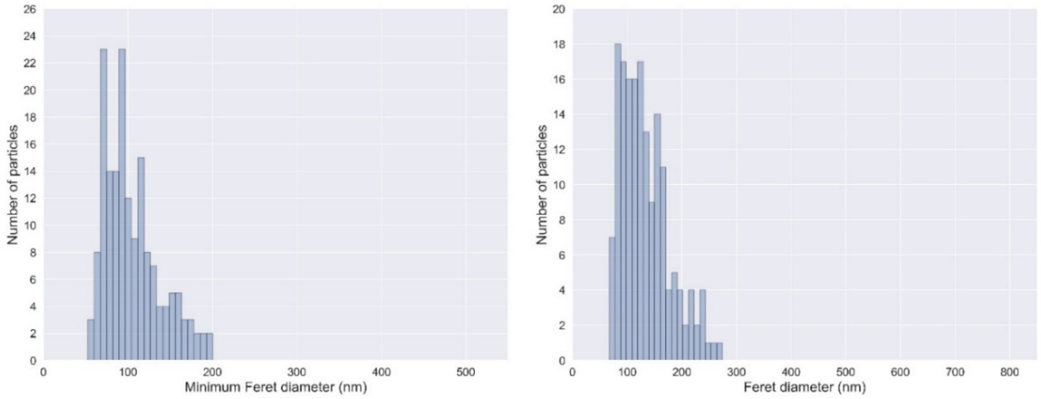

**Supplementary Information 6: Summary of quantitative TEM results.**

**Median values**

| Reference | Layer | Fiber surface area [µm²] | Fiber diameter [µm] | $TiO_2$ agglomerate size | | | | $TiO_2$ constituent particle size | | | |
|---|---|---|---|---|---|---|---|---|---|---|---|
| | | | | Feret min. [nm] | Feret [nm] | Aspect ratio | Number of measured agglomerates | Feret min. [nm] | Feret [nm] | Aspect ratio | Number of measured constituent particles |
| Mask01 | external | 442 | 24 | 197 | 302 | 1.52 | 12 | 131 | 150 | 1.14 | 30 |
| Mask02 | external | 88 | 9 | 123 | 160 | 1.20 | 137 | 125 | 143 | 1.14 | 109 |
| Mask03 | external | 144 | 9 | 188 | 284 | 1.33 | 74 | 124 | 142 | 1.14 | 109 |
| | central | 125 | 11 | 156 | 236 | 1.27 | 75 | 107 | 120 | 1.12 | 102 |
| | internal | 124 | 11 | 173 | 250 | 1.20 | 79 | 133 | 146 | 1.10 | 103 |
| Mask04 | external | 490 | 21 | 220 | 298 | 1.18 | 51 | 182 | 264 | 1.26 | 44 |
| | internal | 435 | 23 | 274 | 449 | 1.59 | 12 | 143 | 158 | 1.11 | 47 |
| Mask05 | external | 396 | 19 | 233 | 315 | 1.29 | 38 | 184 | 224 | 1.22 | 37 |
| | internal | 342 | 18 | 171 | 256 | 1.17 | 61 | 168 | 228 | 1.36 | 55 |
| Mask06 | external | 50 | 7 | 147 | 211 | 1.24 | 59 | 101 | 113 | 1.13 | 109 |
| | central | 940 | 35 | 409 | 597 | 1.33 | 19 | 117 | 132 | 1.12 | 109 |
| Mask07 | external | 93 | 9 | 130 | 187 | 1.25 | 416 | 96 | 113 | 1.18 | 119 |
| | internal | 86 | 9 | 135 | 194 | 1.20 | 291 | 103 | 124 | 1.20 | 94 |
| Mask08 | | 117 | 11 | 184 | 250 | 1.20 | 81 | 99 | 118 | 1.19 | 123 |
| Mask09 | external | 466 | 24 | 450 | 734 | 1.39 | 15 | 147 | 201 | 1.37 | 111 |
| | internal | 415 | 23 | 372 | 625 | 1.38 | 23 | 157 | 220 | 1.40 | 125 |
| Mask10 | external | 102 | 11 | 110 | 164 | 1.20 | 167 | 90 | 115 | 1.28 | 126 |
| Mask11 | external | 95 | 10 | 135 | 194 | 1.29 | 114 | 89 | 118 | 1.33 | 98 |
| Mask12 | external | 170 | 13 | 173 | 226 | 1.18 | 154 | 105 | 132 | 1.26 | 123 |
| | internal | 158 | 12 | 191 | 236 | 1.17 | 123 | 96 | 125 | 1.30 | 166 |

**Mean values**

| Reference | Layer | Fiber surface area [µm²] | Fiber diameter [µm] | $TiO_2$ agglomerate size | | | | $TiO_2$ constituent particle size | | | |
|---|---|---|---|---|---|---|---|---|---|---|---|
| | | | | Feret min. [nm] | Feret [nm] | Aspect ratio | Number of measured agglomerates | Feret min. [nm] | Feret [nm] | Aspect ratio | Number of measured constituent particles |
| Mask01 | external | 402 | 23 | 214 | 326 | 1.58 | 12 | 129 | 143 | 1.11 | 30 |
| Mask02 | external | 88 | 9 | 137 | 158 | 1.28 | 137 | 142 | 168 | 1.18 | 109 |
| Mask03 | external | 140 | 9 | 209 | 318 | 1.41 | 74 | 127 | 152 | 1.20 | 109 |
| | central | 124 | 11 | 186 | 294 | 1.37 | 75 | 113 | 129 | 1.14 | 102 |
| | internal | 123 | 11 | 193 | 276 | 1.30 | 79 | 138 | 156 | 1.13 | 103 |
| Mask04 | external | 488 | 21 | 229 | 322 | 1.28 | 51 | 203 | 296 | 1.46 | 44 |
| | internal | 463 | 24 | 289 | 447 | 1.56 | 12 | 153 | 173 | 1.14 | 47 |
| Mask05 | external | 396 | 18 | 245 | 366 | 1.40 | 38 | 182 | 251 | 1.38 | 37 |
| | internal | 347 | 17 | 187 | 265 | 1.28 | 61 | 174 | 233 | 1.34 | 55 |
| Mask06 | external | 46 | 7 | 163 | 237 | 1.34 | 59 | 105 | 117 | 1.11 | 109 |
| | central | 949 | 35 | 421 | 581 | 1.41 | 19 | 119 | 131 | 1.10 | 109 |
| Mask07 | external | 96 | 9 | 148 | 218 | 1.33 | 416 | 102 | 123 | 1.21 | 119 |
| | internal | 85 | 9 | 154 | 219 | 1.29 | 291 | 110 | 136 | 1.23 | 94 |
| Mask08 | | 116 | 11 | 195 | 273 | 1.28 | 81 | 106 | 129 | 1.22 | 123 |
| Mask09 | external | 469 | 24 | 488 | 747 | 1.60 | 15 | 156 | 228 | 1.46 | 111 |
| | internal | 423 | 23 | 414 | 622 | 1.45 | 23 | 162 | 237 | 1.47 | 125 |
| Mask10 | external | 100 | 11 | 126 | 177 | 1.30 | 167 | 97 | 125 | 1.28 | 126 |
| Mask11 | external | 94 | 10 | 142 | 206 | 1.37 | 114 | 93 | 119 | 1.29 | 98 |
| Mask12 | external | 166 | 13 | 176 | 241 | 1.26 | 154 | 109 | 135 | 1.24 | 123 |
| | internal | 156 | 12 | 197 | 270 | 1.23 | 123 | 105 | 133 | 1.27 | 166 |

**Modal values**

| Reference | Layer | $TiO_2$ agglomerate size | | | | $TiO_2$ constituent particle size | | | |
|---|---|---|---|---|---|---|---|---|---|
| | | Feret min. [nm] | Feret [nm] | Aspect ratio | Number of measured agglomerates | Feret min. [nm] | Feret [nm] | Aspect ratio | Number of measured constituent particles |
| Mask01 | external | 195 | 276 | 1.49 | 12 | 153 | 159 | 1.05 | 30 |
| Mask02 | external | 104 | 140 | 1.13 | 137 | 108 | 122 | 1.10 | 109 |
| Mask03 | external | 154 | 226 | 1.21 | 74 | 126 | 124 | 1.08 | 109 |
| | central | 139 | 228 | 1.24 | 75 | 95 | 114 | 1.06 | 102 |
| | internal | 147 | 203 | 1.15 | 79 | 126 | 136 | 1.06 | 103 |
| Mask04 | external | 208 | 286 | 1.14 | 51 | 169 | 255 | 1.60 | 44 |
| | internal | 252 | 438 | 1.61 | 12 | 136 | 154 | 1.11 | 47 |
| Mask05 | external | 220 | 287 | 1.22 | 38 | 195 | 211 | 1.04 | 37 |
| | internal | 171 | 252 | 1.11 | 61 | 163 | 230 | 1.36 | 55 |
| Mask06 | external | 140 | 180 | 1.13 | 59 | 90 | 95 | 1.06 | 109 |
| | central | 372 | 607 | 1.29 | 19 | 111 | 136 | 1.04 | 109 |
| Mask07 | external | 105 | 139 | 1.14 | 416 | 88 | 97 | 1.18 | 119 |
| | internal | 113 | 160 | 1.09 | 291 | 91 | 107 | 1.16 | 94 |
| Mask08 | | 173 | 241 | 1.14 | 81 | 84 | 98 | 1.14 | 123 |
| Mask09 | external | 419 | 645 | 1.44 | 15 | 144 | 187 | 1.29 | 111 |
| | internal | 344 | 488 | 1.36 | 23 | 149 | 190 | 1.33 | 125 |
| Mask10 | external | 87 | 113 | 1.10 | 167 | 85 | 103 | 1.27 | 126 |
| Mask11 | external | 112 | 165 | 1.19 | 114 | 86 | 114 | 1.24 | 98 |
| Mask12 | external | 128 | 203 | 1.13 | 154 | 90 | 130 | 1.25 | 123 |
| | internal | 173 | 211 | 1.13 | 123 | 87 | 107 | 1.21 | 166 |

**Supplementary information 7: Calculation of the acceptable exposure level to $TiO_2$ by inhalation with uncertainty analysis**

To evaluate whether the amount of titanium dioxide ($TiO_2$) particles at the fiber surface might present a health risk for the wearers of the examined face masks, the mass of $TiO_2$ particles per mask that can be inhaled without adverse effects ($AEL_{mask}$) was estimated. Hereto, the threshold-based approach of ANSES[25] to determine the professional exposure limit to $TiO_2$ nanoparticles was adapted. According to this approach, the occurrence of lung tumors observed in rats is secondary to the production of reactive oxygen species due to the sustained inflammation. Lung inflammation observed after subchronic inhalation exposure of rats to nanoparticles of $TiO_2$ in the Bermudez *et al*[13] study was retained as critical effect. The No Observed Adverse Effect Concentration (NOAEC) has been corrected for human exposure, using the method described in ANSES[25]. Several adjustment factors have been applied. The $AEL_{mask}$ of 3.6 µg has been established assuming an intensive exposure scenario of the general adult population, where two face masks are worn per day of 8 hours.

**Calculation of the acceptable exposure level to $TiO_2$ particles by inhalation, expressed per mask**

| Parameter | Value | Remark |
|---|---|---|
| Critical effect | Lung inflammation | Most sensitive effect |
| Key study | Bermudez et al. | • Uf-$TiO_2$ (Aeroxide $TiO_2$ P25)<br>• Rat – mice – hamsters<br>• 13 weeks, 5d/week, 6h/day<br>• 0, 0.5, 2.0 or 10 mg/m$^3$ |
| Critical dose | NOAEC = 0.5 mg/m$^3$ | No observed adverse effect concentration |
| Adjusted critical dose | $NOAEC_{hec}$ = 0.065 mg/m$^3$ | NOAEC corrected for human exposure |
| Global uncertainty factor | 90 | • Interspecies variability (3)<br>• Intra-human variability (10)<br>• Inadequacy of database (3) |
| AEL | 0.72 µg/m$^3$ | Acceptable exposure limit |
| $AEL_{day}$ | 7.2 µg | AEL per day; 8 hours, 1.25 m$^3$ air inhaled per hour |
| $AEL_{mask}$ | 3.6 µg | AEL per mask; 2 masks per day |

**Uncertainty analysis**

| Sources of uncertainty | Direction[a] |
|---|---|
| **Analytical measurements** | |
| Uncertainty of analytical results | ± |
| **Hazard identification** | |
| Consideration of a threshold mechanism despite concerns regarding possible genotoxicity of $TiO_2$ | - |
| The ‘fiber-grade’ $TiO_2$ (nano)particles present in the examined mask are larger than the nanoparticles evaluated in the reference study | + |
| **Exposure estimations** | |
| Only the (nano)particles that are at the external surface of the fibers of the mask are considered to be susceptible for release | - |
| Only the fraction of the mask in contact with the mouth, the nose and around it will release nanoparticles that may be inhaled | + |
| A limited selection of the many batches, types of fibers and masks on the market has been examined. | ± |
| The calculations are done for the entire masks, assuming no interference of the different layers on particle exposure | + |
| Uncertainty on the level of release of the NP due to lack of data | + |

[a] +: uncertainty with potential to cause overestimation of exposure/risk; -: uncertainty with potential to cause underestimation of exposure/risk.

**Supplementary information 8: Measurement uncertainty of total Ti analysis.**

The in-house expanded measurement uncertainty U (MU) for the method was determined as:

$$U = 2 * u$$

$$u = \sqrt{{u_r}^2 + {u_{between\ days}}^2}$$

With

$$u_r = rsd_{rep}$$

u: the measurement uncertainty of the method
$u_r$: the uncertainty related to the repeatability of the method
$rsd_{rep}$: the relative standard deviation from replicate measurements
the $rsd_{rep}$ was determined for each mask, each different day for two replicate measurements and a pooled $rsd_{rep}$ was calculated to determine the uncertainty related to the repeatability

$$rsd_{rep} = \sqrt{\frac{rsd^2_{rep\ mask1\ day1} + rsd^2_{rep\ mask1\ day2} + rsd^2_{rep\ mask2\ day1} + rsd^2_{rep\ mask2\ day2}}{df}}$$

With

$$u_{between\ days} = \frac{rsd_{days}}{\sqrt{n_{days}}}$$

$u_{between\ days}$: the uncertainty related to the reproducibility of the method
$rsd_{days}$: the between-day standard deviation
$n_{days}$: the amount of replicate days in routine measurements

the $rsd_{days}$ is calculated for each mask from an ANOVA analysis as

$$rsd_{days\ mask\ x} = \frac{\sqrt{\frac{MS_{between\ days} - MS_{within\ days}}{n_{rep}}}}{mean}$$

If $MS_{between\ days}$ was smaller than $MS_{within\ days}$, the $rsd_{days,\ mask\ x}$ was calculated as

$$rsd_{days\ mask\ x} = \sqrt{\frac{MS_{within\ days}}{n}} \sqrt[4]{\frac{2}{N - p}}$$

with n the amount of replicates per day, p the amount of days and N=n*p

a pooled $rsd_{days}$ was calculated to determine the uncertainty

$$rsd_{days} = \sqrt{\frac{rsd^2_{days\ mask1} + rsd^2_{days\ mask2}}{df}}$$

As no reference material nor interlaboratory tests are available, the uncertainty related to the trueness is not included in our in-house measurement uncertainty.
The measurement uncertainty values of both methods are similar, thus for reporting, the value of the most elaborated method (non-woven textiles, u=12%) is used.

Woven textiles, 1 step mineralisation

| | Mask 1 | | Mask 2 | |
|---|---|---|---|---|
| | Day 1 | Day 2 | Day 1 | Day 2 |
| REP 1 | 1086.407 | 918.5746 | 1346.493 | 1575.187 |
| REP 2 | 1060.505 | 917.5852 | 1792.767 | 1442.665 |
| mean | **1073.456** | **918.0799** | **1569.63** | **1508.926** |
| REP 3 | 1062.733 | 898.449 | 1613.762 | 1647.718 |
| REP 4 | 1034.963 | 975.5257 | 1655.258 | 1382.449 |
| mean | **1048.848** | **936.9874** | **1634.51** | **1515.083** |
| total mean per mask | **994** | | **1557** | |

| | | | | |
|---|---|---|---|---|
| $rsd_{rep}$ | 1.6% | 1.4% | 2.9% | 0.3% |
| **$u_r$** | **2%** | | | |

| ***Anova: Single Factor MASK 1*** | | | | | |
|---|---|---|---|---|---|
| ***Source of Variation*** | ***SS*** | ***df*** | ***MS*** | ***F*** | ***P-value*** |
| Between Groups | 35644.000 | 1.000 | 35644.000 | 45.610 | 0.001 |
| Within Groups | 4689.000 | 6.000 | 782.000 | | |
| Total | | | | | |
| $rsd_{days\ mask1}$ | 9.4% | | | | |

| ***Anova: Single Factor MASK 2*** | | | | | |
|---|---|---|---|---|---|
| ***Source of Variation*** | ***SS*** | ***df*** | ***MS*** | ***F*** | ***P-value*** |
| Between Groups | 16200.000 | 1.000 | 16200.000 | 0.652 | 0.450 |
| Within Groups | 149096.000 | 6.000 | 24849.000 | | |
| Total | | | | | |
| $rsd_{days\ mask2}$ | 4% | | | | |
| **$u_{between\ days}$** | **10%** | | | | |
| combined uncertainty | 10% | | | | |
| **U** | **21%** | | | | |

Non-woven textiles, 2 step mineralisation

| | Mask 3 | | Mask 4 | |
|---|---|---|---|---|
| | Day 1 | Day 2 | Day 1 | Day 2 |
| REP 1 | 960.6553 | 1035.196 | 1692.411 | 1817.649 |
| REP 2 | 956.2219 | 1031.499 | 1659.252 | 2347.241 |
| mean | **958.4386** | **1033.348** | **1675.832** | **2082.445** |
| REP 3 | 991.1236 | 1050.903 | 1711.764 | 1939.459 |
| REP 4 | 1019.749 | 1045.381 | 1676.637 | 1860.677 |
| mean | **1005.436** | **1048.142** | **1694.201** | **1900.068** |
| total mean per mask | **1011** | | **1838** | |
| | | | | |
| $rsd_{rep}$ | 3.4% | 1.0% | 0.8% | 6.5% |
| **$u_r$** | **4%** | | | |

| | | | | | |
|---|---|---|---|---|---|
| ***Anova: Single Factor MASK 3*** | | | | | |
| ***Source of Variation*** | ***SS*** | ***df*** | ***MS*** | ***F*** | ***P-value*** |
| Between Groups | 6916.632 | 1.000 | 6916.632 | 14.464 | 0.009 |
| Within Groups | 2869.263 | 6.000 | 478.210 | | |
| Total | | | | | |
| $rsd_{\text{days mask1}}$ | 4.0% | | | | |

| | | | | | |
|---|---|---|---|---|---|
| ***Anova: Single Factor MASK 4*** | | | | | |
| ***Source of Variation*** | ***SS*** | ***df*** | ***MS*** | ***F*** | ***P-value*** |
| Between Groups | 187566.257 | 1.000 | 187566.257 | 6.319 | 0.046 |
| Within Groups | 178103.047 | 6.000 | 29683.841 | | |
| Total | | | | | |
| $rsd_{\text{days mask2}}$ | 11% | | | | |
| **$u_{\text{between days}}$** | **12%** | | | | |
| | | | | | |
| combined uncertainty | 12% | | | | |
| **U** | **24%** | | | | |

**Supplementary Information 9: Illustration of the parameters applied to estimate the fraction of particles at the surface of the fibers.** (A) HAADF-STEM image showing $TiO_2$ particles inside (black arrow) and at the edge (white arrow) of a section of a polyester fiber. (B) A schematic representation of the section of the polyester fiber with agglomerated $TiO_2$ particles (green near-spherical shapes). The thickness of the external ring-shaped surface of the cross-section (green zone), $d_a$, is estimated based on the median value of the minimum Feret diameter distribution of the $TiO_2$ agglomerates.

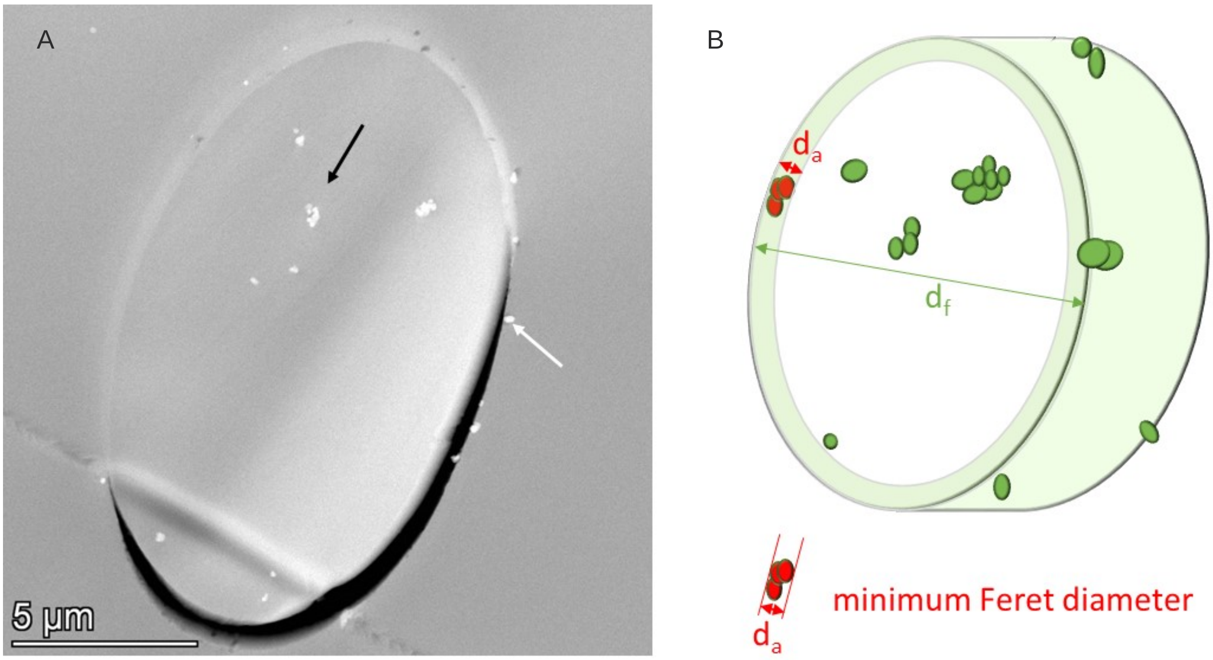

Supplement: Supplementary file 1 — Supplementary Information. [file 41598_2022_6605_MOESM1_ESM.pdf]
